# Overexpression of an *Arabidopsis thaliana* galactinol synthase gene improves drought tolerance in transgenic rice and increased grain yield in the field

**DOI:** 10.1111/pbi.12731

**Published:** 2017-05-03

**Authors:** Michael Gomez Selvaraj, Takuma Ishizaki, Milton Valencia, Satoshi Ogawa, Beata Dedicova, Takuya Ogata, Kyouko Yoshiwara, Kyonoshin Maruyama, Miyako Kusano, Kazuki Saito, Fuminori Takahashi, Kazuo Shinozaki, Kazuo Nakashima, Manabu Ishitani

**Affiliations:** ^1^ International Center for Tropical Agriculture (CIAT) Cali Colombia; ^2^ Tropical Agriculture Research Front (TARF) Japan International Research Center for Agricultural Sciences (JIRCAS) Ishigaki Okinawa Japan; ^3^ Japan Society for the Promotion of Science The University of Tokyo Bunkyo‐ku Tokyo Japan; ^4^ Biological Resources and Post‐harvest Division Japan International Research Center for Agricultural Sciences (JIRCAS) Tsukuba Ibaraki Japan; ^5^ RIKEN Center for Sustainable Resource Science Yokohama Kanagawa Japan; ^6^ RIKEN Center for Sustainable Resource Science Tsukuba Ibaraki Japan; ^7^ Graduate School of Life and Environmental Sciences University of Tsukuba Tsukuba Ibaraki Japan; ^8^ Department of Molecular Biology and Biotechnology Graduate School of Pharmaceutical Sciences Chiba University Chiba Japan

**Keywords:** galactinol synthase, drought, transgenic rice, grain yield, confined field trial

## Abstract

Drought stress has often caused significant decreases in crop production which could be associated with global warming. Enhancing drought tolerance without a grain yield penalty has been a great challenge in crop improvement. Here, we report the Arabidopsis thaliana galactinol synthase 2 gene (*AtGolS2*) was able to confer drought tolerance and increase grain yield in two different rice (*Oryza sativa*) genotypes under dry field conditions. The developed transgenic lines expressing *AtGolS2* under the control of the constitutive maize ubiquitin promoter (*Ubi:AtGolS2*) also had higher levels of galactinol than the non‐transgenic control. The increased grain yield of the transgenic rice under drought conditions was related to a higher number of panicles, grain fertility and biomass. Extensive confined field trials using *Ubi:AtGolS2* transgenic lines in Curinga, tropical japonica and NERICA4, interspecific hybrid across two different seasons and environments revealed the verified lines have the proven field drought tolerance of the Ubi:*AtGolS2* transgenic rice. The amended drought tolerance was associated with higher relative water content of leaves, higher photosynthesis activity, lesser reduction in plant growth and faster recovering ability. Collectively, our results provide strong evidence that *AtGolS2* is a useful biotechnological tool to reduce grain yield losses in rice beyond genetic differences under field drought stress.

## Introduction

Drought is a major abiotic stress condition critically limiting crop production and yield (Edmeades, 2008). Climate prediction models suggest that abiotic stresses will increase in the near future because of global climate change (Ahuja *et al*., 2010). The ever‐rising world population and recurrent global climate change challenge the agricultural system to produce sufficient food to feed the world (Godfray *et al*., 2010). As the world's second‐largest crop, rice plays a critical role in food security for more than half of the world's population (FAO, 2016: http://faostat3.fao.org/browse/Q/QC/E).

Rice accounts for about 27% of total cereal production, with a worldwide production of roughly 738.2 million tons (FAO, 2016). By 2035, a 26% increase in rice production will be required to feed the growing population (Cassman *et al*., 2003; Seck *et al*., 2012). Global water shortage is a major issue for cultivated rice, which needs large quantities of water (Manavalan *et al*., 2012). It was reported that the global reduction in rice production due to drought averages 18 million tons annually (O'Toole, 2004). Worldwide, drought affects approximately 23 million ha of rice production under rainfed conditions. Drought is particularly frequent in unbunded uplands, bunded uplands and shallow rainfed lowland fields in many parts of South and South‐East Asia, sub‐Saharan Africa and Latin America (Serraj *et al*., 2011). To resolve those global problems, it is important to improve crop yields especially within staple food crops like rice (*Oryza sativa* L.) through breeding‐improved stress tolerance.

Transgenic technologies are one of the numerous tools available to plant breeding programmes, which help to open new avenues for crop improvement by developing crop cultivars resistant to various biotic and abiotic stresses (Younis *et al*., 2014). Around 175.2 million hectares of biotech crops were grown globally and transgenic acreage grew 3% in 2013, representing 35% of the global seed market (Marshall, 2014). In rice, progress has been made in the generation and evaluation of transgenic rice events against drought tolerance (Todaka *et al*., 2015).

Plants have evolved several mechanisms to accustom to abiotic stresses through changes at the physiological levels and molecular levels (Todaka *et al*., 2012; Yamaguchi‐Shinozaki and Shinozaki, 2006). It is suggested that overexpression of stress‐related genes could improve drought tolerance in rice (reviewed by Nakashima *et al*., 2014 and Todaka *et al*., 2015). Despite such efforts to develop drought‐tolerant rice plants, very few have been shown to improve grain yields under the field environments (Gaudin *et al*., 2013). Encouraging results include transgenic rice plants expressing *OsNAC5* (Jeong *et al*., 2013), *OsNAC9/SNAC1* (Redillas *et al*., 2012) or *OsNAC10* (Jeong *et al*., 2010), which was shown to improve grain yield under field drought conditions. Many genes that may play an important role under drought have been mostly tested on a single model rice genetic background (Nipponbare) under laboratory conditions, but very few have been tested vigorously in a natural target environment using different commercial rice genetic backgrounds. For improved rice to be accepted by consumers, it is necessary to consider both adaptation to the target environments and fulfilment of local grain quality and taste preferences. This is predominantly important in transgenic studies in which the recipient genetic background is often chosen according to its ability to be transformed rather than agronomic or cultural considerations (Gaudin *et al*., 2013).

The accumulations of metabolite or osmoprotectants are one of the key adaptive mechanisms for plants to handle with dehydration stress and cellular injury (Hare *et al*., 1998). Soluble sugars, including those in the sucrose, trehalose and raffinose families also known as oligosaccharides (RFOs), have been found to accumulate during drought stress in many plants (Collett *et al*., 2004; Farrant, 2007; Peters *et al*., 2007; Taji *et al*., 2002). Galactinol synthase (GolS), a key enzyme in the metabolic pathway leading to RFOs, synthesizes galactinol (from UDP‐Gal and myoinositol), which serves as a galactosyl donor to form raffinose, stachyose and verbascose (Panikulangara *et al*., 2004). It has been reported that the production of enzymes involving the biosynthesis of RFOs, and the resulting accumulation of RFOs, plays critical roles in acquired tolerance of *Arabidopsis thaliana* to drought and heat stresses (Taji *et al*., 2002; Nishizawa *et al*., 2008; reviewed by Sengupta *et al*., 2015). In *Arabidopsis*, seven *GolS* genes and three putative *GolS* genes have been identified, and intricate induction patterns were reported (Nishizawa *et al*., 2008). *AtGolS1* was inducible by drought, salinity (Taji *et al*., 2002) and temperature stresses (Panikulangara *et al*., 2004); *AtGolS2* was induced only by drought and salinity stresses; and *AtGolS3* induction was detected solely after cold stress (Taji *et al*., 2002). Overexpression of *AtGolS2* caused the increase in galactinol and raffinose contents in leaves and exhibited enhanced drought tolerance of transgenic *Arabidopsis* (Taji *et al*., 2002).

Here, we describe the production of transgenic rice events that overexpress the *AtGolS2* cDNA driven by the maize ubiquitin promoter (*Ubi:AtGolS2*) in the background of Curinga (a Brazilian local upland rice variety) and NERICA4 (a popular upland rice variety in African countries) and present the results of multiple confined field trials over two different environmental conditions. Our extensive field test over different seasons and environmental conditions using multiple rice genetic backgrounds clearly demonstrated that *AtGolS2* overexpression consistently increased biomass and grain yield under drought stress conditions. These findings implied that the *Ubi:AtGolS2* transgene played an important role in improving agronomic traits and yield characteristics of rice and that overexpression would be an efficient way to accelerate the rice breeding programme for drought tolerance.

## Results

### Generation and molecular analyses of rice events expressing *Ubi:AtGolS2*

Our goal was to produce and select best‐performing Curinga and NERICA4 transgenic rice lines for drought tolerance by expressing *AtGolS2* from the constitutive maize ubiquitin (*Ubi*) promoter. We accomplished this using *Agrobacterium*‐mediated transformation (Ishizaki and Kumashiro, 2008; Zuniga‐Soto *et al*., 2015). At least 20 independent transgenic events were produced from each variety. T_3_ or T_4_ seeds that possessed genetically fixed single copy of transgene were used for further analyses.

**Figure 1:**
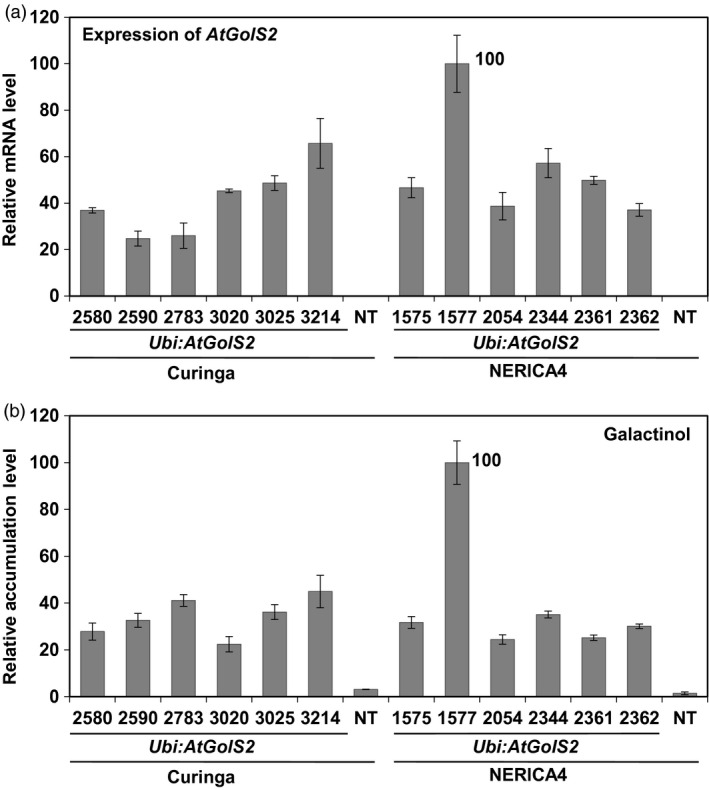
Ectopic overexpression of *AtGolS2* gene confers higher galactinol accumulation on transgenic Curinga and NERICA4 rice. (a) Expression of *AtGolS2* in *Ubi:AtGolS2* transgenic rice was analysed by quantitative real‐time PCR. Expression of *OsUbi1* was analysed as an internal control to normalize the expression of *AtGolS2*. (b) Accumulation of galactinol in *Ubi:AtGolS2* transgenic rice was analysed using GC‐TOF‐MS. The highest average value of the samples was set as 100, and the relative values were shown in (a) and (b). Five plants were combined into one sample for each line, and the relative values are mean ± SD of three technical replicates. Nontransgenic (NT) Curinga and NERICA4 were used as the control.

To link field performance of the transgenic lines to the transgene expression levels and metabolite accumulation levels. Therefore, quantitative PCR expression of *Ubi:AtGolS2* transgenic Curinga and *Ubi:AtGolS2* transgenic NERICA4 events was analysed by quantitative real‐time PCR (RT‐PCR). All of the transgenic Curinga lines expressed the transgene (Figure 1a). Galactinol synthase (GolS) catalyses the first committed step in the biosynthesis of raffinose family oligosaccharides (RFOs) including galactinol and raffinose and plays a key regulatory role in carbon partitioning between sucrose and RFOs (Saravitz *et al*., 1987; Taji *et al*., 2002). We measured galactinol content in the promising transgenic plants grown in glasshouse under unstressed conditions. The NT Curinga and NERICA4 were used as the control. Under normal growth conditions, each transgenic plant showed significantly higher accumulation of galactinol as compared with NT rice plants (Figure 1b). All of the transgenic Curinga lines expressed the transgene and the expression levels of the transgene in lines #3025 and #3214 were higher than in the other lines (#2580, #2590, #2783 and #3020). In case of NERICA4, the *AtGolS2* gene was overexpressed and expression level of the gene and accumulation level of galactinol in line #1577 (NERICA4) were higher than in other lines tested (Figure 1a, b). We also analysed the expression of representative drought marker genes in the *Ubi:AtGolS2* transgenic rice. Expression of genes for a transcription factor OsNAC6, isocitrate lyase (ICL) and late embryogenesis‐abundant protein LEA3 (Maruyama *et al*., 2014; Nakashima *et al*., 2014) was not induced in the events for *Ubi:AtGolS2* without drought stress (Figure S1). These results indicate that the accumulation of galactinol is not related to the expression of these drought‐inducible genes.

### Drought tolerance of *Ubi:AtGolS2* Curinga lines at vegetative‐stage stress

Drought tolerance during the seedling growth period was important for rice plant establishment in areas where early‐season drought overlapped with the vegetative stage. We conducted vegetative‐stage drought experiments using homozygous transgenic rice lines overexpressing *Ubi:AtGolS2* in Curinga. The experiment was conducted in rainout shelter facility at CIAT, Colombia, and biomass was used as a main criterion to select promising lines.

**Figure 2:**
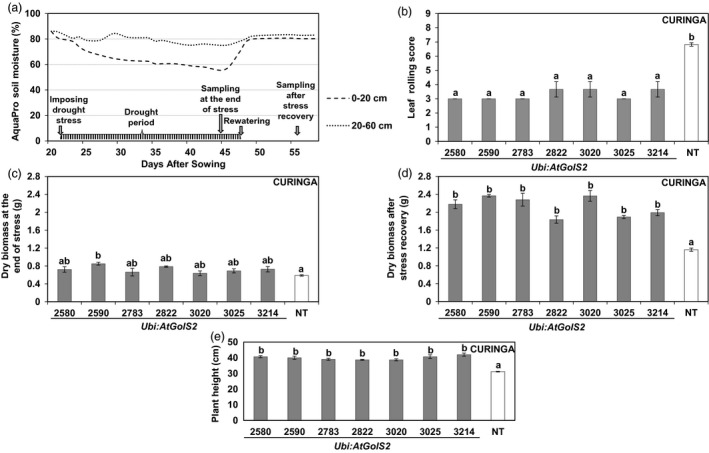
*Ubi:AtGolS2* improves vegetative drought tolerance in transgenic Curinga. (a) Soil moisture profile during the vegetative drought stress rainout shelter experiment. Fragmented line and dotted line indicate upper (0–20 cm) and average lower (20–60 cm) soil moisture, respectively. Arrowheads indicate drought stress scheduling and sampling time. (b) Variation in leaf rolling score among the transgenic lines during peak stress (three weeks after stress). (c) Variation in plant dry biomass among the transgenic lines at the end of the stress. (d) Variation in plant dry biomass among the transgenic lines at the end of the harvest after stress recovery. (e) Variation plant height among the transgenic lines at the end of the stress. Each dry biomass and plant height value represents the mean ± SE (*n* = 3), in each replication data point derived from three individual uniform plants; leaf rolling score based on whole plot performance and represents the mean ± SE (n = 3) from three replications. Different letters in each column denote significant differences at *P *<* *0.05 by Tukey–Kramer method.

To evaluate the growth performance of the *Ubi:AtGolS2*‐overexpressing Curinga under drought conditions at the vegetative stage, three‐week‐old transgenic and nontransgenic (NT) Curinga control plants were subjected to drought stress for up to 3 weeks in November‐December 2011 (Figure 2a). Agronomic data collected before stress treatment showed that there was no significant variation among the lines, which helps to explain the uniformity in the experiment.

During peak stress, the transgenic lines significantly maintained more plant height than NT Curinga (Figure 2e). The NT Curinga started to show visual symptoms of drought‐induced leaf rolling at an earlier stage than the transgenic plants (Figure 2b). The transgenic lines showed low leaf rolling score, 2‐3 compared with the NT Curinga plants with (6.5) during peak drought stress (Figure 2b). Only the event #2590 accumulated significantly higher dry biomass than NT Curinga at the end of drought stress (Figure 2c). However, dry biomasses of the transgenic plants measured after rewatering were significantly higher than those of NT Curinga in all but one instance (Figure 2d).

### Drought tolerance of the *Ubi:AtGolS2* Curinga lines under Managed Drought Stress Environment (MDSE)

In order to confirm the drought tolerance of Curinga transgenic lines at the reproductive stage, three consecutive confined field trials were conducted under the removable rainout shelter facility at CIAT, Palmira. As mentioned above, we had conducted two drought stress trials in the year 2012 over two cultivating seasons (2012‐rainy season‐MDSE‐Trial‐1 and 2012‐dry season‐MDSE‐Trial‐2) and one in 2014 (2014‐rainy season‐MDSE‐Trial) (Figure S2). Up to eight independent T_4_ single‐copy homozygous transgenic and NT Curinga lines were used to conduct the dry‐down experiments. In addition to the field drought experiments, one normal well‐watered paddy field trial (WW‐field trial) was also conducted during the dry season of 2012 (Table S1). In the WW‐field trial, single plant grain yield of all the tested transgenic lines and NT Curinga was not significantly different (Table S1), which suggests no yield penalty in these transgenic lines. Based on these results, grain yield was also used as main parameter to compare yield in dry fields between these transgenic lines and NT Curinga.

**Figure 3:**
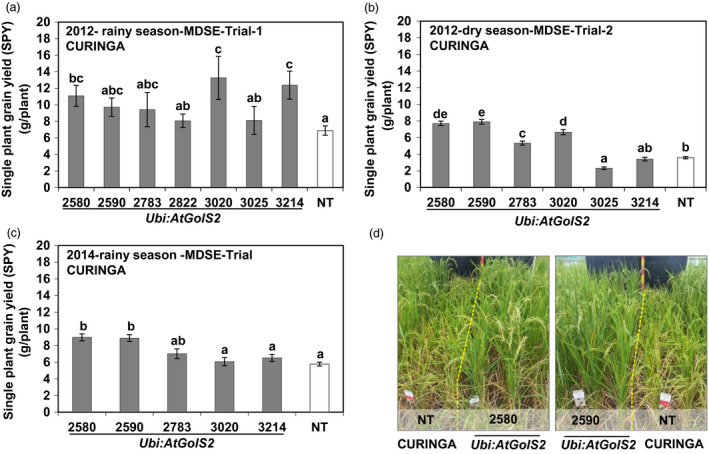
*Ubi:AtGolS2* improves rice grain yield in Managed Drought Stress Environment (MDSE) over the growing seasons—rainout shelter experiments, CIAT, Palmira. Single plant grain yield performance of Curinga transgenic lines in 2012‐rainy season‐MDSE‐Trial‐1 (a), 2012‐dry season‐MDSE‐Trial‐2 (b) and 2014‐rainy season‐MDSE‐Trial (c). (d) Field performance of NT Curinga and promising transgenic lines in 2014‐rainy‐MDSE‐Trial. Photographs were taken next day after rewatering for recovery after drought stress at the flowering stage. Each single plant yield value represents the mean ± SE (*n* = 9–24). Different letters in each column denote significant differences at *P *<* *0.05 by Tukey–Kramer method.

Drought intensity varied among drought trials from mild to severe (Figure S2). Although similar levels of stress duration and environmental conditions occurred in the rainy season drought experiments, it was observed that average single plant yield of NT Curinga was sharply reduced compared to the other two rainy season experiments (Figure 3a, b and c). This suggests a dry season effect. Additionally, we found that soil moisture rapidly decreased within the 0‐ to 40‐cm soil layer compared to other two rainy season experiments (Figure S2b). The level of drought stress imposed under upland rainout shelter conditions was equivalent to that which caused an average reduction of around 60%–70% in the single plant grain yield obtained in the NT Curinga under well‐watered paddy field conditions (Table S1 and Figure 3a, b and c).

Statistical analysis of yield and yield‐related parameters scored for three rainout shelter drought experiments revealed that the *Ubi:AtGolS2* overexpression consistently produced grain yield compared to NT across the season. Interestingly, in the *Ubi:AtGolS2* Curinga lines, the morphophysiological trait performance was significantly better than NT Curinga (Table S2 and Figure 3). In each trial, we found some transgenic lines that had significantly higher yield: three of eight transgenic lines in 2012‐rainy‐MDSE‐Trial‐1, four of six transgenic lines in 2012‐dry‐MDSE‐Trial‐2 and two of five transgenic lines in 2014‐rainy‐MDSE‐Trial, respectively (Figure 3a, b and c). We also found two promising transgenic lines (#2580 and #2590), which consistently outperformed NT Curinga in terms of grain yield over the drought experiments (Figure 3). In these lines, the higher grain yield under severe drought stress (2012‐dry‐MDSE‐Trial‐2) was associated with a significantly higher number of panicles, higher accumulation of biomass, low leaf rolling and leaf drying score, faster recovering ability, early flowering (Table S2), panicle length and grain fertility. Furthermore, under moderate stress (drought experiments in rainy season), no significant difference in panicle number and biomass between transgenic and nontransgenic lines was observed. Altogether, these results demonstrate that the *Ubi:AtGolS2* expression increased grain yield under drought stress conditions imposed at the reproductive stage through a mechanism that involves the maintenance of early flowering, increased vegetative biomass, higher numbers of panicles and enhanced grain fertility (Table S2).

### Analysis of physiological parameters in *Ubi:AtGolS2* Curinga lines

Based on the results of the rainout shelter experiments in 2012 (the 2012‐MDSE‐Trial‐1 and 2), five promising T_4_ *Ubi:AtGolS2* Curinga lines were selected for further analysis of physiological parameters and field gene expression analysis in the 2014‐rainy‐MDSE‐Trial. During the dry‐down experiment, the most uniform transgenic lines for physiological analysis were tagged and repeated measurements were taken during drought stress.

**Figure 4:**
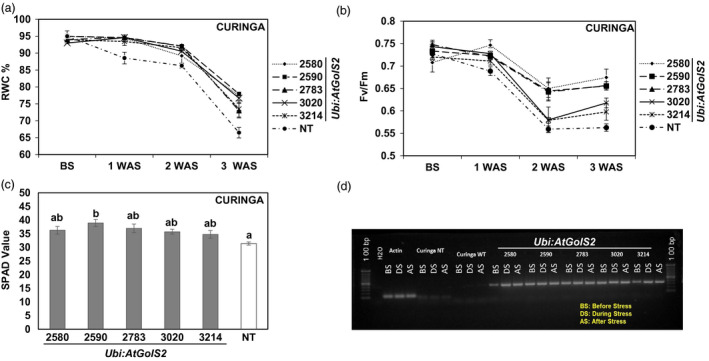
Variation in physiological parameters among the Curinga *Ubi:AtGolS2* transgenic lines in 2014‐rainy‐MDSE‐Trial. (a) Percentage of relative water content (RWC) values of transgenic and NT lines at before, first, second and third weeks after stress (WAS). (b) Changes in chlorophyll fluorescence ( *F*_*V*_ */* *F*_*M*_ ) of transgenic and NT lines at before (BS), first, second and third weeks after stress (WAS). (c) SPAD chlorophyll values of transgenic and NT lines at peak drought stress. (d) RT‐PCR analysis of *Ubi:AtGolS2* transgenic and NT lines evaluated at different timing points: before stress (BS), during peak stress (DS) and after stress (AS). RT‐PCR analyses were performed using RNAs from leaf tissue at different points of drought development using *Ubi:AtGolS2* gene‐specific primers. Each physiological parameter value represents the mean ± SE (*n* = 9), three individual plants from three replications. Different letters in each figure denote significant differences at *P *<* *0.05 by Tukey–Kramer method.

Relative water content (RWC) was measured before and after subjecting the transgenic lines and NT Curinga to the drought stress treatment. Before stress, there were no obvious differences in the leaf RWC between NT Curinga and transgenic lines, and the RWC was within the range of 92%–95% (Figure 4a). After the lines were subjected to water stress for one week, the RWC of the NT Curinga leaves reduced sharply with respect to their first reading (before stress) from 95% to 89 %, whereas the RWC of most of the transgenic lines declined very slowly (#2580, 94%; #2590, 94%; #2783, 94%; #3020, 94%; and #3214, 93%). After three weeks of drought stress, the RWC of the transgenic lines had declined by just 18%–22% as compared to 30% in the NT Curinga. Lines #2580, #2590, #2783, #3020 and #3214 maintained RWC very well even three weeks after drought stress, with RWC percentages of 73, 77, 77, 72 and 76, respectively. The rapid decline of the RWC (average of 66%) was observed in the NT Curinga after three weeks of drought stress.

To further verify the mechanism of drought tolerance, we measured *F*_*v*_/*F*_*m*_ values of the transgenic and NT Curinga during stress period using FluorPen‐FP100, (Photon Systems Instruments, spol. s r.o., Czech Republic) (Figure 4b). The *F*_*v*_/*F*_*m*_ values represent the maximum photochemical efficiency of photo system (PS) II in a dark‐adapted state, where *F*_*v*_ stands for variable fluorescence and *F*_*m*_ stands for maximum fluorescence. Initially under unstressed conditions, the *F*_*v*_/*F*_*m*_ values of both NT and transgenic plants were similar, ranging from 0.70 to 0.74. After one week of drought stress, the *F*_*v*_/*F*_*m*_ value of the NT Curinga slightly decreased (0.68), but we did not find any significant differences between NT Curinga and the *Ubi:AtGolS2* transgenic lines. However, after three weeks of drought stress, the *F*_*v*_/*F*_*m*_ value for the transgenic lines #3214, #2580, #2590, #2783 and #3020 was maintained at 0.59, 0.67, 0.65, 0.65 and 0.61, respectively, while the NT Curinga value significantly decreased to 0.56 (Figure 4b). The promising transgenic lines #2580 and #2590 showed significantly higher *F*_*v*_/*F*_*m*_ values compared to NT Curinga even after three weeks of stress.

Chlorophyll content was measured using a SPAD‐502 Chlorophyll Meter (Konica Minolta Inc., Tokyo, Japan). Transgenic and NT Curinga plants were measured for their chlorophyll content before and during peak stress (Figure 4c). Before stress, the SPAD values of the transgenic and NT Curinga plants were not significantly different ranging from 37 to 40. At peak stress (three weeks after stress), the chlorophyll values of the NT Curinga plants were reduced (average of 34) compared to the initial reading; in contrast, promising lines like #2580 and #2590 maintained a similar chlorophyll content after three weeks of the stress (Figure 4c). A RT‐PCR analysis was also performed on the tested promising lines in the 2014‐rainy‐MDSE‐Trial during different stages of the stress development to confirm the expression of the *Ubi:AtGolS2* transgene under field conditions (Figure 4d).

### Drought tolerance of *Ubi:AtGolS2* Curinga in the different environments—Target Environment (TE) Trial

**Figure 5:**
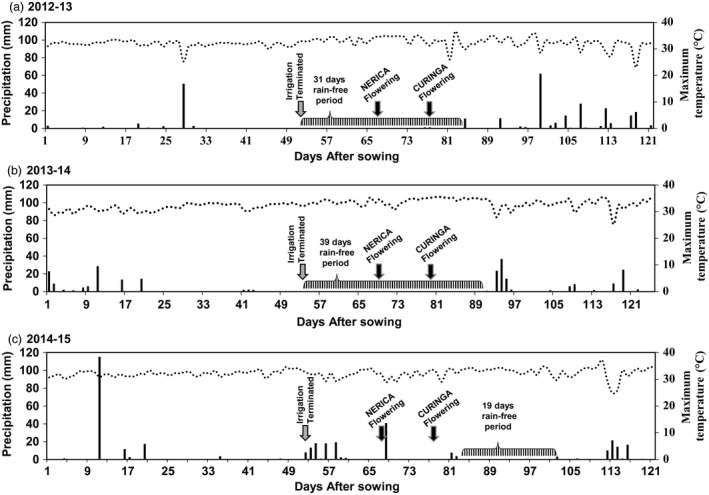
Rainfall and temperature pattern during crop period in upland confined rainfed field trial, CIAT, Santa Rosa upland station. (a) Climatic profile of TE‐2012‐13‐Trial. (b) Climatic profile of TE‐2013‐14‐Trial. (c) Climatic profile of TE‐2014‐15‐Trial. Black bar shows amount of rainfall received during crop period, and dotted line graph shows the maximum daily temperature during trial period. Temperature data are daily averages and rainfall is daily total.

Based on the initial vegetative and reproductive drought stress experiments, up to six potential transgenic Curinga lines along with the NT Curinga were chosen for upland rainfed field trials at CIAT Santa Rosa station, Villavicencio, Colombia. To test the hypothesis of gene × environment interactions of *Ubi:AtGolS2*, the three consecutive TE field trials were carried out from 2012 to 2015. The field trial conditions, design and the plot size were well described in the experimental procedure section. The rainfall and temperature pattern of this site during trial period is shown in Figure 5. Ten years of rainfall data from this site revealed that natural rainfall failure events usually occur in the months of January–February, which coincides with the reproductive stage of the crop. For instance, trial years TE‐2012‐13 and TE‐2013‐14 were very dry with continuous rain‐free days of 31 and 39, respectively (Figure 5a and b). However, trial year TE‐2014‐15 had rainfall on and off during the reproductive stage (Figure 5c).

**Figure 6:**
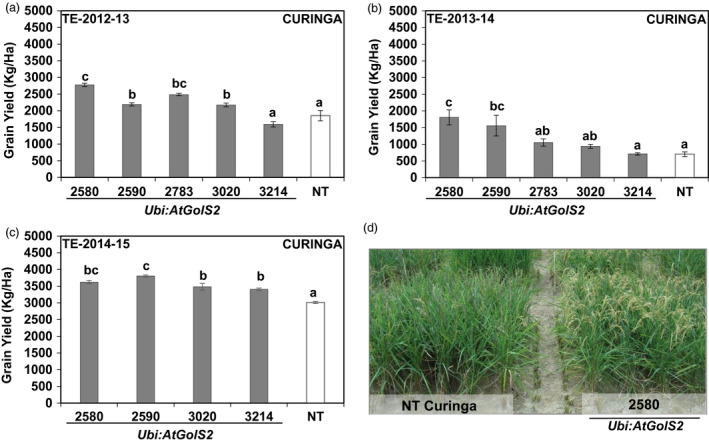
*Ubi:AtGolS2* improves Curinga grain yield in Target Environment (TE)—Santa Rosa rainfed Trial, CIAT upland rainfed station, Villavicencio. (a) Grain yield performance of Curinga transgenic lines in TE‐2012‐13‐Trial. (b) Grain yield performance of Curinga transgenic lines in TE‐2013‐14‐Trial. (c) Grain yield performance of Curinga transgenic lines in TE‐2014‐15‐Trial. (d) Field performance of NT Curinga and promising transgenic event 2580 at TE‐2013‐14‐Trial. Photographs were taken during stress at the grain‐filling stage. Estimated grain yield (kg/ha) was derived from plot yield from three replications, and value represents the mean ± SE (*n* = 3). Different letters in each column denote significant differences at *P *<* *0.05 by Tukey–Kramer method.

In the first two rainfed trials (TE‐2012‐13 and TE‐2013‐14), Curinga transgenic lines reached 50% flowering significantly earlier than NT Curinga (4 and 5 days earlier), indicating that *Ubi:AtGolS2* overexpression induced earliness in Curinga lines (Table S3). The continuous rain‐free period during the reproductive stage caused a marked reduction in soil moisture in the 0‐ to 40‐cm soil layer (Figure S3). Under these severe stress conditions, lines #2580 and #2590 maintained higher panicle number and showed significantly higher grain yield (GY) with relative gains of 49%, 18%, 34% and 17%, respectively, compared to NT Curinga (Figure 6a and Table S3) in the first trial (TE‐2012‐13). We also observed yield gains continued in promising transgenic lines in the second (TE‐2013‐14) and third (TE‐2014‐15) field trials (Figure 6b and c). In the third rainfed trial (TE‐2014‐15), drought stress was mild; there was about 19 days rain‐free period that coincided the grain‐filling stage (Figure 5c). Field vegetative performance of *Ubi:AtGolS2* transgenic Curinga (#2580) was much better than NT Curinga as shown in Figure 6d. Thus, the average grain yields of promising *Ubi:AtGolS2* transgenic lines were significantly increased compared to NT Curinga in three field trials (Figure 6).

### Drought tolerance in the different genetic background expressing *Ubi:AtGolS2*

To understand how the *Ubi:AtGolS2* ubiquitously works in a rice genotype, we conducted drought tolerance experiments in homozygous transgenic lines overexpressing *Ubi:AtGolS2* in the interspecific hybrid, NERICA4. We first tested seedling survival rate in a glasshouse pot experiment at Japan International Research Center for Agriculture Sciences (JIRCAS) in Japan. Seedling survival of NERICA4 transgenic lines was evaluated through a previously reported method (Ishizaki *et al*., 2013). Before drought stress treatment, no obvious phenotypic differences were observed between the NT NERICA4 plants and the *Ubi:AtGolS2* transgenic NERICA4 lines. After nine days of drought treatment and subsequent recovery for seven days, the majority of NT NERICA4 never recovered and only 11.5% survived. By contrast, four of seven *Ubi:AtGolS2* transgenic NERICA4 lines exhibited a significantly higher survival ratio, ranging from 26.2% to 34.5% (Table S4). These results demonstrate that *Ubi:AtGolS2* can significantly improve seedling survival under drought in NERICA4.

**Figure 7:**
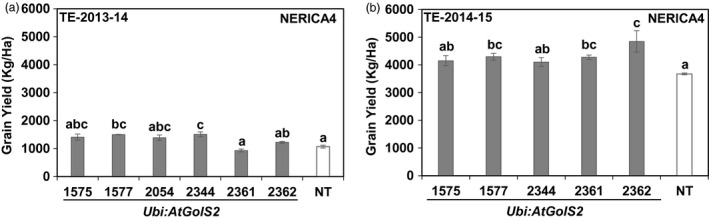
*Ubi:AtGolS2* improves NERICA4 grain yield in rainfed upland trials. (a) Grain yield performance of NERICA4 lines in Target Environment (TE)‐2013‐14‐Trial at Santa Rosa rainfed trial. (b) Grain yield performance of NERICA4 lines in TE‐2014‐15‐Trial at Santa Rosa rainfed trial. Estimated grain yield (kg/ha) derived from plot yield. Estimated grain yield (kg/ha) was derived from plot yield from three replications, and value represents the mean ± SE (*n* = 3). Different letters in each column denote significant differences at *P *<* *0.05 by Tukey–Kramer method.

The *Ubi:AtGolS2*‐NERICA4 lines were also evaluated during the second (TE‐2013‐14) and third (TE‐2014‐15) rainfed reproductive trials in Colombia along with Curinga lines; NERICA4 lines were not included in the trial 1 (TE‐2012‐13) (Figure 7 and Table S5). AquaPro soil moisture profiles indicated that the conditions of NERICA4 plots were similar to those of Curinga plots in TE‐2013‐14‐Trial and TE‐2014‐15‐Trial (Figure S4 a and b). Under severe drought stress conditions in the second trial (TE‐2013‐14), the *Ubi:AtGolS2*‐NERICA4 lines #1577 and #2344 showed significantly higher GY with relative gains of 34% and 49%, respectively, compared to NT NERICA4 (Figure 7a). In the third trial (TE‐2014‐15), #1577, #2361 and #2362 showed significantly higher grain yield than NT NERICA4 (Figure 7b). Interestingly, line 1577 consistently performed well in both vegetative and reproductive experiments (Table S4 and Figure 7).

### Correlation between accumulation level of galactinol and grain yield

To link field performance of the transgenic lines to the expression level of transgene *AtGolS2* and the galactinol accumulation levels, we calculated Pearson's coefficient of correlation between accumulation level of galactinol and mRNA level of *AtGolS2*, SPY and GY in Curinga and NERICA4 evaluated under field (Figure S5). The expression level of *AtGolS2* correlated with the accumulation level of galactinol: Pearson's coefficient of correlation between those factors was 0.72 in Curinga and 0.94 in NERICA4, revealing that the expression of *AtGolS2* certainly conferred the accumulation of galactinol in rice plant. However, SPY and GY did not always correlate with the accumulation level of galactinol: Pearson's coefficient of correlation between those factors ranged from ‐0.05 to 0.65, suggesting the galactinol did not have dose effects on yield under field.

## Discussion

### *Ubi:AtGolS2* is versatile: improving drought tolerance across stages of rice, genetic background, drought intensity and environments

While developing drought‐tolerant crops, plant productivity should be taken into consideration. Plant productivity is widely affected by natural drought incidences under field conditions (Todaka *et al*., 2015). Droughts are random events and dry spells can occur at virtually any time during the rice growing period in drought‐prone areas, leading to drought stress of varying intensity. Although rice is highly sensitive to drought stress during the reproductive stage (Venuprasad *et al*., 2007), drought at early vegetative stage of rice growth can considerably affect plant performance. Commonly, drought survival test during the vegetative stage is obtained under laboratory or glasshouse conditions and is therefore not perfectly comparable to interpretations made under real‐field conditions. Extensive field trials are thus critical for the appropriate evaluation of stress‐tolerant transgenic crops (Todaka *et al*., 2015). In this paper, we carried out field trials in CIAT, Colombia, and demonstrated that *Ubi:AtGolS2* overexpression in rice was effective at conferring drought tolerance during both the vegetative and the reproductive stages (Figures 2 and 3). It is a very rare phenomenon when the results obtained from vegetative screening experiments concur with reproductive stage, indicating that the *Ubi:AtGolS2* overexpression can be exploited for both early‐ and mid‐season drought. This is very important in the perspective of targeting rice varieties to rainfed environments where rainfall uncertainty is expected.

In rice, several reports are available that examine field drought tolerance caused by overexpression of transgenes (Xiao *et al*., 2009; You *et al*., 2012; Yu *et al*., 2013). However, most of these studies were conducted on plants that were grown under glasshouse conditions. There have been instances where a transgene‐mediated trait expressed in the glasshouse was unstable under field conditions (Brandle *et al*., 1995). As it was reported that the effect of transgene expression in wheat varied from year to year based on the climatic conditions of a particular growing season (Bahieldin *et al*., 2005), it was considered essential to explore the yield stability of the *Ubi:AtGolS2* transgenic rice in different seasons, for example rainy and dry and under several environmental conditions such as rainfed and well‐watered conditions.

In this study, higher GY in the *Ubi:AtGolS2* transgenic lines was consistently observed over the season and environments (Figures 3, 6 and 7). The promising *Ubi:AtGolS2* transgenic lines showed significantly enhanced drought tolerance in the field across different genetic backgrounds, Curinga and NERICA4, with a grain yield of 17%–100% higher than NT Curinga under mild to severe drought stress, whereas the transgenic lines displayed no significant differences under normal growth conditions (Figures 3, 6, 7 and Table S1). These improvements of grain yields under drought can be considered greater than what has been reported for other transgenic rice lines expressing genes conferring field drought tolerance, which have often been challenged with milder stresses as demonstrated by the grain yield reduction under drought of the control checks (Oh *et al*., 2009). However, in this study, the drought intensity was mild because plants were irrigated to evade leaf rolling, which resulted in a yield loss of around 32% in the WT. In another study, rice plants overexpressing *OsNAC10* showed enhanced drought tolerance during the flowering stage and increased grain yield by 25%–42% compared to WT, but again milder drought stress conditions were applied (Jeong *et al*., 2010).

A limited number of studies applied field drought conditions similar to our work (Todaka *et al*., 2015). Under severe stress, rice transgenic lines expressing *OsCPI1* showed 2.5‐ to 3‐fold greater GY over the control, for which yield dropped 90% (Huang *et al*., 2007). Likewise, plants overexpressing *LOS5* and *ZAT10* exhibited gains between 11% and 36% compared to their controls which suffered 82% yield reduction (Xiao *et al*., 2009).

Through this international collaborative project, we realized the importance of conducting the initial screening efforts in a farmer‐adapted variety, because these are popular over large growing areas, locally adapted and because relatively quick introgression of the transgene into other megavarieties is possible (Gaudin *et al*., 2013). In our study, we attempted to improve two farmer‐adapted varieties, one from Latin America (Curinga) and another one from Africa (NERICA4). The phenotype might be controlled by genes (**G**) including transgenes and genetic background (genotypes) in transgenic plants, and plant responses to drought are also influenced by environment (**E**) including intensity, duration and frequency of the stress as well as by diverse plant–soil–atmosphere interactions (Saint Pierre *et al*., 2012). It is always suggested to test **G **× **G** and **G **× **E** interactions/stability before recommending a potential transgene into the breeding pipeline. In this study, we have conducted drought experiments in two contrasting field seasons (rainy and dry). We found the response of the *Ubi:AtGolS2* lines to be different than the NT control based on the season. However, regardless of the season, the promising lines #2580 and #2590 had significantly greater plant biomass and panicle numbers. A similar result was found within the target environment.

Our results provide strong evidence that overexpression of *AtGolS2* is a useful biotechnological tool to reduce yield losses under field drought conditions under different environmental conditions (**E**) and in different rice genetic backgrounds (**G**), which suggests that *AtGolS2* is an essential gene to improve drought tolerance in rice regardless of **G** × **G** and **G** × **E** interactions.

### Mechanism of drought tolerance offered by the *Ubi:AtGolS2* transgene

Even though many stress resistance genes have been identified in noncrop species such as *Arabidopsis*, evaluation of the effect of these genes on improving field drought tolerance in a given crop has seldom been reported (Xiao *et al*., 2009). GolS plays a key role in the accumulation of galactinol under abiotic stress conditions, conferring drought stress tolerance to plants, because galactinol may function as osmoprotectants and scavenger of hydroxyl radicals (Nishizawa *et al*., 2008; Taji *et al*., 2002). Based on our extensive evaluation of transgenic lines, the *Ubi:AtGolS2* transgene improved grain yield of rice under drought conditions. Although high expression of *AtGolS2* and accumulation of galactinol were confirmed in *Ubi:AtGolS2* events, no significant change in the expression of drought marker genes was induced (Figure 1 and Figure S1). These results suggest that drought tolerance of *Ubi:AtGolS2* transgenic lines with the accumulation of galactinol was not correlated with the expression of drought‐responsive genes. This could be contributed through the following mechanisms. First, the transgenic lines are more tolerant to drought and gain yield over the NT because they are protected by elevated galactinol (RFOs) that can act as osmoprotectants and scavenger of hydroxyl radicals (Figure 1b). The increased transcription of *GolS* genes during drought has been reported in many plants and crops. In C*ucumis melo*, it was observed that GolS activates accumulation of RFO in plants submitted to drought stresses (Volk *et al*., 2003).

As second physiological perspective, the promising Curinga transgenic lines have a better maximum photochemical efficiency (*F*_*v*_/*F*_*m*_) and leaf chlorophyll content than NT under drought stress (Figure 4b). The decrease in *F*_*v*_ */F*_*m*_ and SPAD chlorophyll values under drought stress could be an indicator of oxidative stress and damage in PSII (Farooq *et al*., 2009). Under severe drought stress, we observed high SPAD chlorophyll and *F*_*v*_/*F*_*m*_ values in the *Ubi:AtGolS2* Curinga than in NT Curinga, and the *Ubi:AtGolS2* leaves were greener than those of the NT, which confirmed normal photosynthesis in transgenic *Ubi:AtGolS2* rice. In addition to photosynthetic‐related traits, stress‐related traits such as RWC, leaf rolling and drying of transgenic lines were significantly better than NT Curinga (Tables S2, S3 and S5). Maintenance of high plant water status, as expressed in high RWC of the *Ubi:AtGolS2* rice, was an good indicator of drought tolerance (as shown by Babu *et al*., 2004), and capacity of transgenic lines maintained higher leaf RWC compared with NT Curinga under drought stress, which was consistent with their ability to postpone dehydration (as indicated by Castonguay and Markhart, 1992). In this study, we observed that the *Ubi:AtGolS2* transgenic lines had higher RWC values than NT rice during peak drought stress and had a lower leaf rolling and leaf drying score (Figure 4a; Tables S2, S3 and S5). By contrast, we observed that NT rice quickly wilted and dried as compared to those transgenic lines with higher RWC values under drought stress.

Third, the *AtGolS2* Curinga transgenic lines showed earlier flowering than NT Curinga (Table S3) under drought stress, but displayed no difference in growth under normal growth conditions (Table S1). In rice, early maturation is an escape mechanism to ensure production under conditions of stress (Gur *et al*., 2010). In this study, the *Ubi:AtGolS2* Curinga lines showed the earliest flowering and exhibited higher grain fertility (82%) than NT (70%), which may be due to the drought escape mechanism of transgenic lines (data not shown). However, early flowering was not observed in the *Ubi:AtGolS2* NERCA4 background (Table S5). As NERICA4 is a short‐duration variety, early flowering of the *Ubi:AtGolS2* lines may be profound in Curinga due to the long duration nature.

Transcription levels of the *AtGolS2* gene of the transgenic lines correlated with the accumulation level of galactinol; however, the accumulation level of galactinol did not correspond with their field performance (Figures 1 and S5). These results suggest that good field performance might not always be associated with levels of gene expression and of accumulation of galactinol. The complexities of environments and other factors influencing performance of rice plants under drought may be reasons for no dosage effects of galactinol on grain yield under field.

## Conclusions and prospects

Our study reported extensive field evaluation of transgenic rice plants expressing the *Ubi:AtGolS2* transgene under drought stress environments under field conditions in Colombia. We clearly observed that the *Ubi:AtGolS2* expression and the accumulation of galactinol significantly enhanced grain yield under drought field conditions, but did not affect either grain yield or plant growth under well‐watered paddy field conditions. Improved grain yield under stress was associated with early flowering, higher biomass accumulation, higher number of panicles and lower panicle sterility.

In this study, the same gene construct, the *Ubi:AtGolS2,* transgene was tested on two different commercial genetic backgrounds, Curinga and NERICA4, and contrasting different seasons and different environments. We presented the results of extensive confined field testing of transgenic rice overexpressing *AtGolS2* and the responses of these transgenic rice plants to contrasting environments. Notably, we evaluated the agronomic traits of these transgenic lines at all stages of plant growth in the field as a function of the environment and genetic background. As the *Ubi:AtGolS2* transgene was tested in the commercial rice genetic backgrounds of Latin America (Curinga) and Africa (NERICA4), we think it is easy to pyramid the *Ubi:AtGolS2* transgene into ongoing transgenic rice breeding programmes in Latin America and Africa. The promising NERICA4 transgenic lines selected from this study can be integrated into ongoing NEWEST—the NERICA4 (Nitrogen‐use Efficient, Water‐use Efficient and Salt Tolerant) rice project where extensive transgenic field trials are currently being implemented in Ghana, Uganda and Nigeria through USAID feed the future programme. Development of this drought‐tolerant rice through the *Ubi:AtGolS2* transgene should have significant economic and environmental benefits in low‐input agricultural systems like Latin America and Africa.

## Experimental procedures

### Generation of *Ubi:AtGolS2* Plants

To generate transgenic rice plants overexpressing *AtGolS2* encoding galactinol synthase 2 of *Arabidopsis thaliana* (Taji *et al*., 2002), the pBIG‐ubi vector was used (Becker, 1990; Ito *et al*., 2006). *AtGolS2* cDNA was amplified using *Bam*HI linker primers. The resulting DNA fragment carrying *Bam*HI sites at the 5′ and 3′ termini was inserted into pBIG‐ubi at the *Bam*HI site. The construct was introduced into rice cv. Curinga and NERICA4 by *Agrobacterium*‐mediated transformation as described previously (Ishizaki and Kumashiro, 2008; Zuniga‐Soto *et al*., 2015). The molecular characterization of putative transgenic events involved PCR and Southern blot analysis. The primers used for this study are reported in Table S6.

### Expression analysis of rice plants expressing *Ubi:AtGolS2*

The transgenic and nontransgenic (NT) rice plants (Curinga and NERICA4) were grown in soil‐filled, open‐bottomed 50‐mL plastic tubes in the glasshouse. After the drought treatment, the leaves from five plants were collected, frozen in liquid nitrogen and stored at −80 °C. Total RNA was isolated from the leaf samples using RNAiso Plus reagent (Takara Bio, Shiga, Japan). Extracted RNA was subjected to a DNase treatment using a RQ1 DNase (Promega, WI), and complementary DNA was synthesized using a PrimeScript RT Master Mix (Takara Bio). Real‐time quantitative RT‐PCR was performed with the QuantStudio 7 Flex real‐time PCR system (Thermo Fisher Scientific, MA) using SYBR Premix Ex Taq (Takara Bio). Primers used for qRT‐PCR are listed in Table S6.

### Sugar metabolite analysis of rice plants expressing *Ubi:AtGolS2*

The transgenic and control rice lines were grown in soil‐filled, open‐bottomed 50‐ml plastic tubes in the glasshouse. After the drought treatment, the leaves from five plants were collected, frozen in liquid nitrogen and stored at −80 °C. Sugar metabolites were analysed using GC‐TOF‐MS as described previously by Maruyama *et al*. (2014).

### Seedling survival test of NERICA4 transgenic events

The ability of NERICA4 transgenic events to survive under rapid drying was evaluated by the reported method (Ishizaki *et al*., 2013).

### Vegetative drought stress experiment in confined field

To evaluate the drought tolerance of transgenic rice plants at vegetative stage, single‐copy independent homozygous lines of *Ubi:AtGolS2* Curinga transgenic lines, together with nontransgenic (NT) Curinga controls, were direct‐seeded in confined field conditions under a rainout shelter at the International Center for Tropical Agriculture (CIAT), Palmira, Colombia, in the dry season, November‐December 2011. A randomized block design was employed with three replicates with each event sown in two rows placed 16 cm apart in a rainout shelter where the depth of restructured soil was 85 cm. Each row was 1 m long and 40 plants were accommodated in each row with equal spacing (5 cm) between plants. Drought stress was imposed by withholding irrigation at initial tillering stage (21 days after direct sowing) and rewatered after 21 days (3 weeks) until severe wilting symptoms appeared in NT Curinga (Figure 2a). The intensity of drought was monitored through AquaPro soil moisture probes (AquaPro sensors Inc, California, USA). Plant height, the number of tillers and destructive plant dry biomass data were measured from uniform tagged plants at the before, during peak stress and at the end of the harvest after rewatering. Leaf rolling was determined at the time of peak drought stress.

### Managed Drought Stress Environment (MDSE) Trial—Rainout Shelter (RS) reproductive Stage trial at CIAT, Palmira, Colombia

All rainout shelter reproductive stress experiments were carried out at our confined field facility at CIAT, Palmira, Colombia. For Curinga, three confined field drought trials (from 2012 to 2014) over two contrasting seasons were conducted under the movable semi‐automatic rainout shelter facility. All three experiments were conducted using same protocol with respect to designs and field drought characterization. A randomized block design with three replications was followed to layout the experiment under the rainout shelter facility at CIAT. The seeds of T_4_ homozygous lines were sown in the dry soil of the experimental plots in rows (20 cm spacing between rows). Each event was sown in two rows placed 20 cm apart where the depth of restructured soil was 85 cm. Each row was 2 m long and had 20 plants with equal spacing. The recommended fertilizer application for upland rice was used. Drought was imposed by withholding irrigation when panicle initiation was around 10 mm long (60–66 days after sowing in the case of Curinga) for 3–4 weeks (or) until severe leaf rolling and drying appeared in the NT control. Then, the plants were rewatered to 90% field capacity until physiological maturity. The intensity of drought was monitored through AquaPro soil moisture probes that were installed to measure moisture in the soil profile to a depth of 0.85 m.

Leaf rolling (LR), leaf drying (LD) and drought recovery scores were recorded on a 1‐9 IRRI scale standardized for rice (IRRI, 2002). The following agronomic traits were measured based on the criteria established in the Standard Evaluation System for Rice (SES) (IRRI, 2002): flowering date, plant height (cm), single plant dry biomass (g), panicle length (cm), the number of tillers, the number of fully emerged panicles and grain fertility (%). Single plant yield (from five more uniform tagged plants from each block with three replications) and plot yield were also recorded.

The degree of relative chlorophyll content in the fully expanded flag leaf was determined while the plant was under stress, using a SPAD‐502 Chlorophyll Meter (Konica Minolta Co., Tokyo, Japan). Chlorophyll a fluorescence parameters were also measured using a FluorPen FP100 chlorophyll (Photon Systems Instruments, spol. s r.o., Czech Republic). Relative water content was calculated using the protocol based on Schonfeld *et al*. (1988).

### Well‐watered experiment in confined field conditions

To evaluate the yield components of the transgenic Curinga lines under normal well‐watered field conditions, selected independent T_4_ homozygous lines of *Ubi:AtGolS2* transgenic plants together with NT controls were transplanted to a rice paddy confined field at CIAT, Palmira (dry season, September–January 2013). A randomized design was employed with three replicates of two 2‐m‐long rows per plot. For each plot, 20 seedlings per line were randomly transplanted with a 20 × 10 cm spacing 25 days after sowing. The recommended fertilizer application for lowland rice was used. Yield parameters were scored for five tagged uniform plants per plot.

### RT‐PCR analysis of field samples—Rainout shelter Drought trials

Total RNA was extracted from the flag leaves of tested promising transgenic lines at before stress, peak stress and after rewatering from the 2014‐rainy‐MDSE‐rainout shelter trial using the Trizol reagent (Invitrogen‎). Reverse transcription was carried out using DNase (Promega) and SuperScript III (Invitrogen). Endpoint PCR was conducted with primers listed in Table S6, using standard protocols and an annealing temperature of 55 °C. PCR products were checked on 1% agarose gel with SYBR‐safe stain.

### Target Environment (TE) Trial—Confined Field Evaluation of Transgenic lines at Santa Rosa, Colombia

To evaluate yield components of transgenic plants under rainfed upland conditions with natural drought condition, the most promising independent T_4_ homozygous lines of Curinga and NERICA4 from the previous drought experiments along with their NT controls were evaluated in a replicated plot trial with randomized block design from 2013 to 2015 (three consecutive field trials) at CIAT Santa Rosa rainfed upland station, Colombia. Promising NERICA4 lines were selected based on the survival test results from Japan and rainout shelter trials. An upland field trial was laid out in a random complete block design with three replicates. The transgenic events along with NT rice were sown in 2 × 2 m plots with 25 × 10 cm spacing. Seeds were sown directly by hand at the rate of 120 kg/ha when soil moisture was about 80% of field capacity. The recommended fertilizer application for upland rice was used. The plants were established in dry soil and irrigation was provided until 50 days after sowing (DAS) via sprinklers to establish the crop. After plant establishment, irrigation was stopped and plants were totally dependent on rainfall. The soil moisture was monitored throughout the cropping period by Aquapro soil moisture device. Plant growth and development of each of the transgenic events relative to NT rice was monitored regularly and plot yield (g) was recorded. Grain yield (kg/ha) was estimated from plot yield based on plant density.

## Data analysis

Data were analysed by one‐factor ANOVA at *P *<* *0.05. When significant differences were found, multiple comparisons by the Tukey–Kramer method (*P *<* *0.05) were made.

## Author contributions

K.S., K.N. and M.I designed the total experiments. M. G. S., S.O. and M.O.V. planned, conducted and analysed drought field experiments. B.D. and M.I. conducted transformation experiments at CIAT. T.I. conducted transformation experiments and glasshouse experiments at JIRCAS. T.O. and K.N. conducted gene expression experiments. K.Y., K. M., M. K. and K. S. conducted sugar analysis. F.T. made the construct for transformation. M.G.S. wrote the manuscript, and all the authors checked it.

## Ethical standards

The authors declare that the transgenic experiments comply with the current biosafety laws of the country in which they were performed.

## Supporting information


**Figure S1** Expression analysis of drought responsive genes in *Ubi:AtGolS2* transgenic rice.
**Figure S2** Aquapro soil moisture profile and work schedules of Curinga plots during rainout shelter managed drought stress environment (MDSE) trial, CIAT, Palmira.
**Figure S3** Aquapro soil moisture profile and work schedules of Curinga plots during Target environment (TE) trial, CIAT, Santa Rosa upland rainfed station.
**Figure S4** Aquapro soil moisture profile and work schedules of NERICA4 plots during Target Environment (TE) trial, CIAT, Santa Rosa upland rainfed station.
**Figure S5** Pearson's correlation coefficient between accumulation level of galactinol and mRNA level of AtGolS2, single plant yield (SPY), and grain yield (GY) in Curinga and NERICA4 evaluated under field.

**Table S1** Agronomic data capture from 2012 well‐watered paddy field trial at CIAT, Palmira, using *Ubi:AtGolS2* Curinga lines.
**Table S2** Agronomic data capture from rainout‐shelter trial (2012‐rainy‐MDSE‐Trial‐1, 2012‐dry‐MDSE‐Trial‐2, and 2014‐rainy‐Trial) at CIAT, Palmira using *Ubi:AtGolS2* Curinga transgenic lines.
**Table S3** Agronomic data capture from Santa Rosa target Environment (TE) upland trial using *Ubi:AtGolS2* Curinga lines.
**Table S4** The survival rates of NT NERICA4, *Ubi:AtGolS2* NERICA4 lines under drought stress.
**Table S5** Agronomic data capture from Santa Rosa target environment (TE) trial using *Ubi:AtGolS2* NERICA4 lines.
**Table S6** Primer sequences used in this study.
