# Supplementary material for: Overexpression of an Arabidopsis thaliana galactinol synthase gene improves drought tolerance in transgenic rice and increased grain yield in the field
Source: Plant Biotechnol J. 2017 May 3;15(11):1465–77. doi: 10.1111/pbi.12731 (PMC5633756; doi:10.1111/pbi.12731)
Supplement: Supplementary file 1 — Figure S1 Expression analysis of drought responsive genes in Ubi:AtGolS2 transgenic rice. Figure S2 Aquapro soil moisture profile and work schedules of Curinga plots during rainout shelter managed drought stress environment (MDSE) trial, CIAT, Palmira. Figure S3 Aquapro soil moisture profile and work schedules of Curinga plots during Target environment (TE) trial, CIAT, Santa Rosa upland rainfed station. Figure S4 Aquapro soil moisture profile and work schedules of NERICA4 plots during Target Environment (TE) trial, CIAT, Santa Rosa upland rainfed station. Figure S5 Pearson's correlation coefficient between accumulation level of galactinol and mRNA level of AtGolS2, single plant yield (SPY), and grain yield (GY) in Curinga and NERICA4 evaluated under field. [file PBI-15-1465-s002.pptx]

## Slide 1
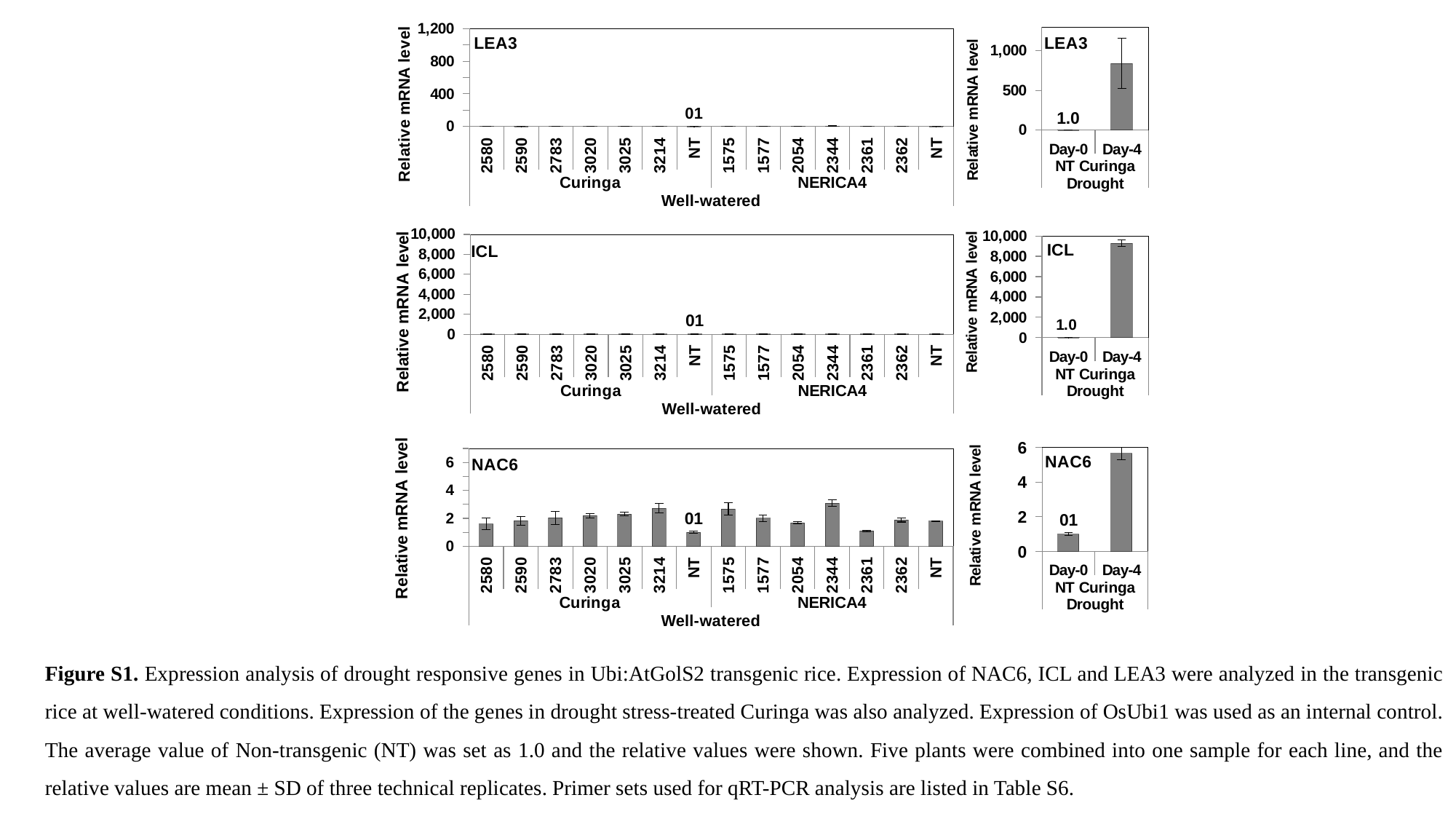

### Chart:
| Category | |
|---|---|
| Day-0 | 1.0 |
| Day-4 | 840.9991936727114 |
### Chart:
| Category | |
|---|---|
| 2580 | 0.0 |
| 2590 | 1.272598719956505 |
| 2783 | 0.0 |
| 3020 | 1.859612673178988 |
| 3025 | 0.0 |
| 3214 | 0.0 |
| NT | 1.0 |
| 1575 | 0.0 |
| 1577 | 2.336134453968979 |
| 2054 | 2.408568805119994 |
| 2344 | 3.708972935222316 |
| 2361 | 0.0 |
| 2362 | 0.0 |
| NT | 1.345695608343859 |
### Chart: ICL
| Category | |
|---|---|
| Day-0 | 1.0 |
| Day-4 | 9259.423739297863 |
### Chart: ICL
| Category | |
|---|---|
| 2580 | 1.96908110485824 |
| 2590 | 0.336703799228883 |
| 2783 | 0.302395207491833 |
| 3020 | 0.0198063504033299 |
| 3025 | 0.996306101406338 |
| 3214 | 3.291128150744673 |
| NT | 1.0 |
| 1575 | 0.834356566845602 |
| 1577 | 1.202784177726616 |
| 2054 | 0.0945564327563604 |
| 2344 | 0.205903476125272 |
| 2361 | 0.192795450385894 |
| 2362 | 1.088005915091556 |
| NT | 0.683695883183903 |
### Chart:
| Category | |
|---|---|
| Day-0 | 1.0 |
| Day-4 | 5.667385873786925 |
### Chart:
| Category | |
|---|---|
| 2580 | 1.599993502469372 |
| 2590 | 1.820189188414373 |
| 2783 | 2.032502536946049 |
| 3020 | 2.184786188686797 |
| 3025 | 2.310671992540937 |
| 3214 | 2.719808054893889 |
| NT | 1.0 |
| 1575 | 2.676770035354428 |
| 1577 | 2.006420813469636 |
| 2054 | 1.676186216051724 |
| 2344 | 3.088826149339789 |
| 2361 | 1.090246604120784 |
| 2362 | 1.87325715525968 |
| NT | 1.802171555788877 |Figure S1. Expression analysis of drought responsive genes in Ubi:AtGolS2 transgenic rice. Expression of NAC6, ICL and LEA3 were analyzed in the transgenic rice at well-watered conditions. Expression of the genes in drought stress-treated Curinga was also analyzed. Expression of OsUbi1 was used as an internal control. The average value of Non-transgenic (NT) was set as 1.0 and the relative values were shown. Five plants were combined into one sample for each line, and the relative values are mean ± SD of three technical replicates. Primer sets used for qRT-PCR analysis are listed in Table S6.

## Slide 2
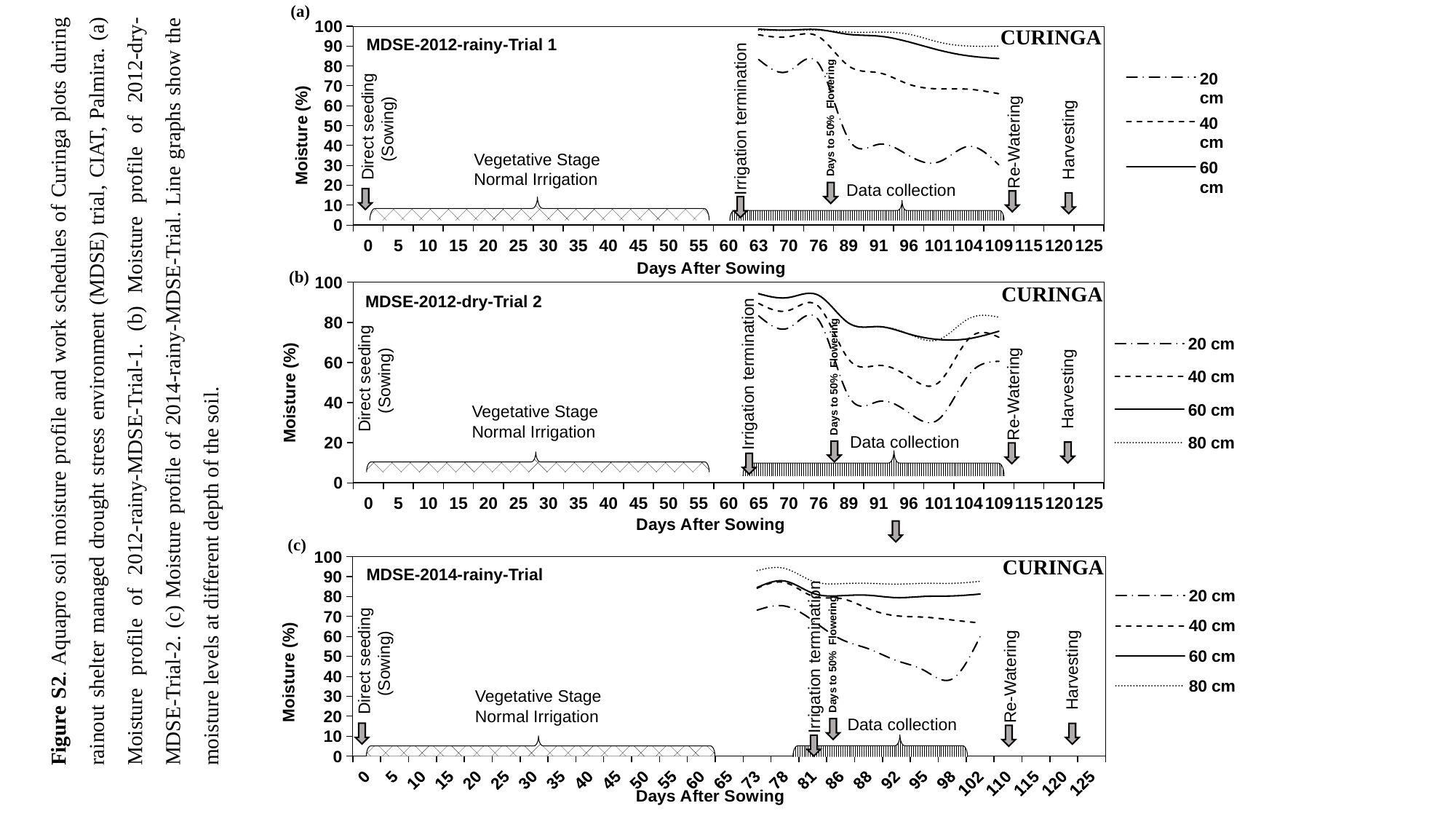

(a)
### Chart
| Category | 20 cm | 40 cm | 60 cm | 80 cm |
|---|---|---|---|---|
| 0 | None | None | None | None |
| 5 | None | None | None | None |
| 10 | None | None | None | None |
| 15 | None | None | None | None |
| 20 | None | None | None | None |
| 25 | None | None | None | None |
| 30 | None | None | None | None |
| 35 | None | None | None | None |
| 40 | None | None | None | None |
| 45 | None | None | None | None |
| 50 | None | None | None | None |
| 55 | None | None | None | None |
| 60 | None | None | None | None |
| 63 | 83.29268292682924 | 95.70731707317073 | 98.52439024390245 | 98.0 |
| 70 | 77.35722784057108 | 94.70374776918501 | 98.01859012492561 | 98.0 |
| 76 | 81.16049382716048 | 94.75308641975307 | 98.27160493827162 | 98.0 |
| 89 | 43.17283950617284 | 80.01234567901236 | 95.90123456790126 | 97.0 |
| 91 | 40.58024691358025 | 76.59259259259255 | 95.02469135802465 | 97.0 |
| 96 | 34.97530864197531 | 70.82716049382717 | 92.09876543209874 | 96.0 |
| 101 | 31.69135802469136 | 68.48148148148148 | 88.0 | 92.0 |
| 104 | 39.5206263173743 | 68.28635953026195 | 85.04878048780485 | 90.0 |
| 109 | 30.28395061728395 | 66.03703703703704 | 83.71604938271606 | 90.0 |
| 115 | None | None | None | None |
| 120 | None | None | None | None |
| 125 | None | None | None | None |CURINGA
MDSE-2012-rainy-Trial 1
Direct seeding (Sowing)
Irrigation termination
Days to 50% Flowering
Re-Watering
Harvesting
Vegetative Stage
Normal Irrigation
Data collection
### Chart
| Category | 20 cm | 40 cm | 60 cm | 80 cm |
|---|---|---|---|---|
| 0 | None | None | None | None |
| 5 | None | None | None | None |
| 10 | None | None | None | None |
| 15 | None | None | None | None |
| 20 | None | None | None | None |
| 25 | None | None | None | None |
| 30 | None | None | None | None |
| 35 | None | None | None | None |
| 40 | None | None | None | None |
| 45 | None | None | None | None |
| 50 | None | None | None | None |
| 55 | None | None | None | None |
| 60 | None | None | None | None |
| 65 | 83.29268292682924 | 89.5 | 94.36280487804875 | 94.36280487804875 |
| 70 | 77.28395061728395 | 85.9876543209877 | 92.45061728395062 | 92.45061728395062 |
| 76 | 81.16049382716048 | 87.95679012345673 | 93.5185185185185 | 93.5185185185185 |
| 89 | 43.17283950617284 | 61.5925925925926 | 79.67901234567898 | 79.67901234567898 |
| 91 | 40.58024691358025 | 58.58641975308639 | 77.9320987654321 | 77.9320987654321 |
| 96 | 34.97530864197531 | 52.90123456790122 | 74.27469135802465 | 74.27469135802465 |
| 101 | 31.69135802469136 | 50.08641975308639 | 71.46604938271606 | 71.46604938271606 |
| 104 | 53.9506172839506 | 71.9166666666667 | 71.9166666666667 | 81.9166666666667 |
| 109 | 60.63 | 72.63 | 75.63 | 82.63 |
| 115 | None | None | None | None |
| 120 | None | None | None | None |
| 125 | None | None | None | None |(b)
CURINGA
MDSE-2012-dry-Trial 2
Figure S2. Aquapro soil moisture profile and work schedules of Curinga plots during rainout shelter managed drought stress environment (MDSE) trial, CIAT, Palmira. (a) Moisture profile of 2012-rainy-MDSE-Trial-1. (b) Moisture profile of 2012-dry-MDSE-Trial-2. (c) Moisture profile of 2014-rainy-MDSE-Trial. Line graphs show the moisture levels at different depth of the soil.
Direct seeding (Sowing)
Irrigation termination
Days to 50% Flowering
Harvesting
Re-Watering
Vegetative Stage
Normal Irrigation
Data collection
(c)
### Chart
| Category | 20 cm | 40 cm | 60 cm | 80 cm |
|---|---|---|---|---|
| 0 | None | None | None | None |
| 5 | None | None | None | None |
| 10 | None | None | None | None |
| 15 | None | None | None | None |
| 20 | None | None | None | None |
| 25 | None | None | None | None |
| 30 | None | None | None | None |
| 35 | None | None | None | None |
| 40 | None | None | None | None |
| 45 | None | None | None | None |
| 50 | None | None | None | None |
| 55 | None | None | None | None |
| 60 | None | None | None | None |
| 65 | None | None | None | None |
| 73 | 73.17999999999998 | 84.18518518518518 | 84.53846153846148 | 93.0 |
| 78 | 75.23076923076921 | 87.0 | 87.74074074074076 | 94.07407407407405 |
| 81 | 67.92592592592592 | 80.14814814814815 | 81.66666666666667 | 87.66666666666667 |
| 86 | 58.66666666666664 | 79.03703703703704 | 80.4074074074074 | 86.44444444444447 |
| 88 | 53.77777777777778 | 73.85185185185176 | 80.62962962962963 | 86.62962962962963 |
| 92 | 47.8888888888889 | 70.33333333333327 | 79.44444444444447 | 86.22222222222223 |
| 95 | 42.92592592592595 | 69.6666666666667 | 80.07407407407405 | 86.62962962962963 |
| 98 | 38.7037037037037 | 68.2222222222222 | 80.2962962962963 | 86.59259259259255 |
| 102 | 59.92592592592595 | 66.7407407407407 | 81.25925925925927 | 87.62962962962963 |
| 110 | None | None | None | None |
| 115 | None | None | None | None |
| 120 | None | None | None | None |
| 125 | None | None | None | None |CURINGA
MDSE-2014-rainy-Trial
Direct seeding (Sowing)
Irrigation termination
Days to 50% Flowering
Harvesting
Re-Watering
Vegetative Stage
Normal Irrigation
Data collection

## Slide 3
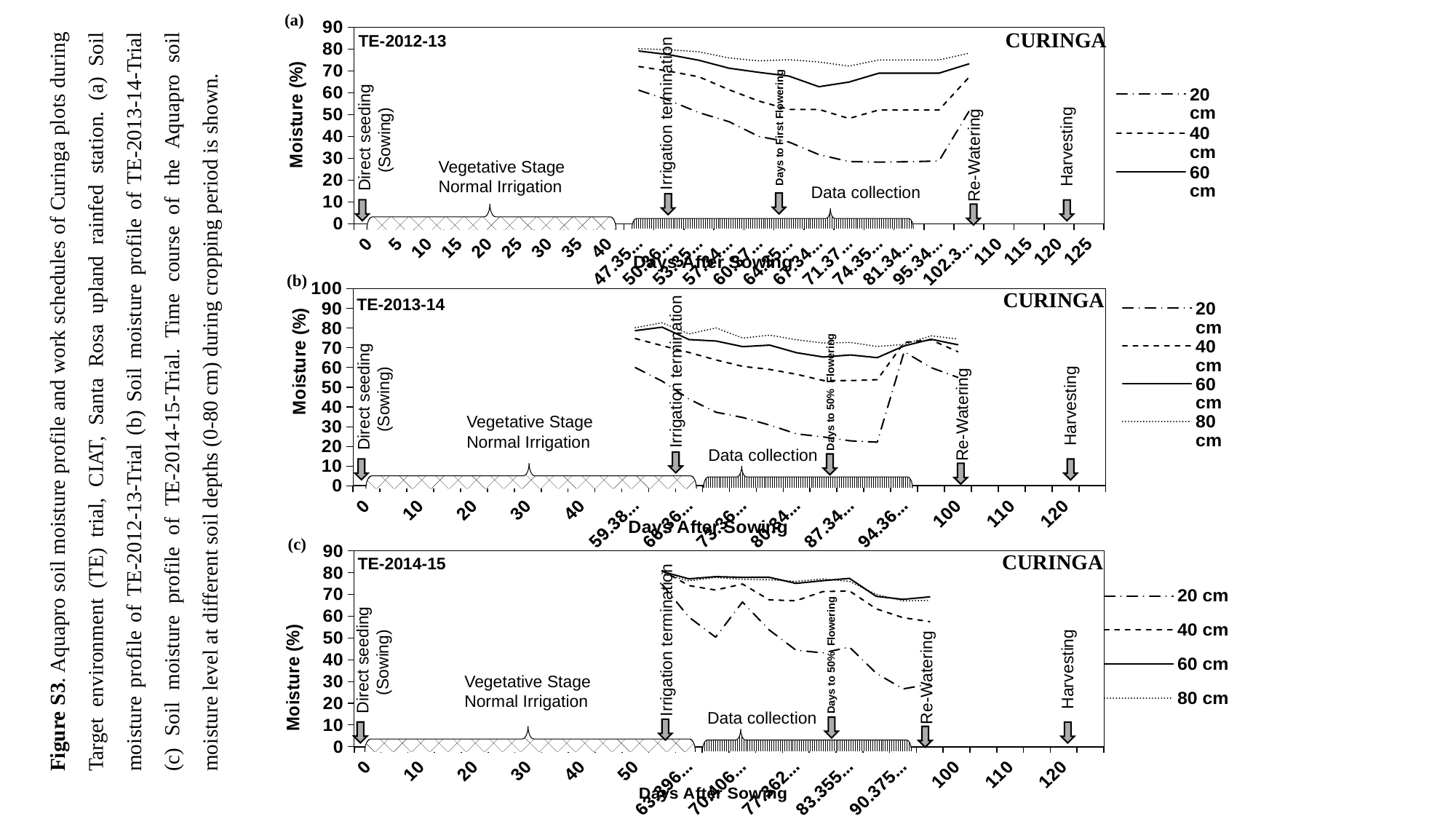

### Chart
| Category | 20 cm | 40 cm | 60 cm | 80 cm |
|---|---|---|---|---|
| 0 | None | None | None | None |
| 5 | None | None | None | None |
| 10 | None | None | None | None |
| 15 | None | None | None | None |
| 20 | None | None | None | None |
| 25 | None | None | None | None |
| 30 | None | None | None | None |
| 35 | None | None | None | None |
| 40 | None | None | None | None |
| 47.356909722220728 | 61.12903225806452 | 71.96774193548384 | 79.06451612903228 | 80.12903225806446 |
| 50.365150462959733 | 56.51612903225806 | 69.8709677419355 | 77.38709677419355 | 79.67741935483868 |
| 53.35447916666449 | 50.87096774193547 | 67.258064516129 | 74.83870967741935 | 78.7096774193548 |
| 57.344560185185387 | 46.74193548387097 | 61.38709677419353 | 71.22580645161288 | 75.90322580645159 |
| 60.371550925927302 | 39.96774193548387 | 56.16129032258065 | 69.32258064516128 | 74.58064516129032 |
| 64.350081018521379 | 37.41935483870968 | 52.35483870967739 | 67.61290322580646 | 75.09677419354836 |
| 67.345960648148335 | 31.67741935483871 | 52.35483870967739 | 62.74193548387097 | 74.06451612903228 |
| 71.373113425928764 | 28.48387096774193 | 48.19354838709678 | 64.8709677419355 | 72.16129032258061 |
| 74.354062500002328 | 28.25806451612902 | 52.09677419354838 | 68.93548387096774 | 75.0 |
| 81.343912037038507 | 28.41935483870968 | 52.09677419354838 | 68.93548387096774 | 75.0 |
| 95.343912037038507 | 28.77419354838709 | 52.09677419354838 | 68.93548387096774 | 75.0 |
| 102.34391203703851 | 51.41935483870968 | 67.03225806451613 | 73.19354838709674 | 78.06451612903228 |
| 110 | None | None | None | None |
| 115 | None | None | None | None |
| 120 | None | None | None | None |
| 125 | None | None | None | None |(a)
CURINGA
TE-2012-13
Irrigation termination
Direct seeding (Sowing)
Days to First Flowering
Harvesting
Re-Watering
Vegetative Stage
Normal Irrigation
Data collection
### Chart
| Category | 20 cm | 40 cm | 60 cm | 80 cm |
|---|---|---|---|---|
| 0 | None | None | None | None |
| 5 | None | None | None | None |
| 10 | None | None | None | None |
| 15 | None | None | None | None |
| 20 | None | None | None | None |
| 25 | None | None | None | None |
| 30 | None | None | None | None |
| 35 | None | None | None | None |
| 40 | None | None | None | None |
| 45 | None | None | None | None |
| 59.382060185183953 | 60.0 | 74.7083333333333 | 78.75 | 80.25 |
| 62.40296296296583 | 53.08333333333334 | 71.12499999999999 | 80.5 | 82.7083333333333 |
| 66.360243055554719 | 44.0 | 67.66666666666667 | 74.2083333333333 | 77.0833333333333 |
| 70.352581018516446 | 37.33333333333334 | 63.875 | 73.5 | 80.12499999999999 |
| 73.360300925924079 | 34.66666666666664 | 60.58333333333334 | 70.62499999999999 | 74.9583333333333 |
| 77.350682870368445 | 30.79166666666667 | 59.04166666666662 | 71.37499999999999 | 76.4583333333333 |
| 80.341898148144509 | 26.33333333333331 | 56.58333333333334 | 67.5416666666667 | 74.12499999999999 |
| 84.353344907409294 | 24.75 | 53.41666666666662 | 65.3333333333333 | 72.4583333333333 |
| 87.344131944446417 | 22.79166666666667 | 53.41666666666662 | 66.4166666666667 | 72.75 |
| 91.352627314816317 | 22.125 | 53.83333333333334 | 65.0416666666667 | 70.7083333333333 |
| 94.367372685184804 | 68.0416666666667 | 72.75 | 71.0 | 71.75 |
| 97.844282407408173 | 60.0 | 74.0416666666667 | 74.37499999999999 | 76.12499999999999 |
| 100 | 55.0 | 68.0416666666667 | 71.5833333333333 | 74.5 |
| 105 | None | None | None | None |
| 110 | None | None | None | None |
| 115 | None | None | None | None |
| 120 | None | None | None | None |
| 125 | None | None | None | None |(b)
CURINGA
TE-2013-14
Figure S3. Aquapro soil moisture profile and work schedules of Curinga plots during Target environment (TE) trial, CIAT, Santa Rosa upland rainfed station. (a) Soil moisture profile of TE-2012-13-Trial (b) Soil moisture profile of TE-2013-14-Trial (c) Soil moisture profile of TE-2014-15-Trial. Time course of the Aquapro soil moisture level at different soil depths (0-80 cm) during cropping period is shown.
Irrigation termination
Direct seeding (Sowing)
Days to 50% Flowering
Harvesting
Re-Watering
Vegetative Stage
Normal Irrigation
Data collection
(c)
### Chart
| Category | 20 cm | 40 cm | 60 cm | 80 cm |
|---|---|---|---|---|
| 0 | None | None | None | None |
| 5 | None | None | None | None |
| 10 | None | None | None | None |
| 15 | None | None | None | None |
| 20 | None | None | None | None |
| 25 | None | None | None | None |
| 30 | None | None | None | None |
| 35 | None | None | None | None |
| 40 | None | None | None | None |
| 45 | None | None | None | None |
| 50 | None | None | None | None |
| 58.464236111110949 | 74.61904761904762 | 81.0 | 80.57142857142854 | 79.6 |
| 63.396203703705403 | 59.61904761904759 | 74.0952380952381 | 77.19047619047613 | 76.25 |
| 66.389687499999113 | 50.38095238095238 | 72.0476190476191 | 78.19047619047613 | 77.8 |
| 70.406145833330811 | 66.5238095238095 | 74.80952380952381 | 77.85714285714285 | 77.0 |
| 73.392314814816899 | 53.71428571428572 | 67.5238095238095 | 77.9047619047619 | 76.75 |
| 77.362638888887858 | 44.42857142857143 | 67.0952380952381 | 75.0952380952381 | 76.0 |
| 80.365902777775744 | 43.14285714285715 | 71.3333333333333 | 76.3333333333333 | 77.1 |
| 83.35519675925751 | 45.80952380952381 | 71.61904761904762 | 77.38095238095238 | 76.05 |
| 86.37549768518511 | 33.80952380952381 | 63.38095238095238 | 69.1428571428571 | 70.05 |
| 90.375092592592353 | 26.61904761904762 | 59.33333333333334 | 67.7619047619048 | 67.05 |
| 93.365335648144509 | 28.76190476190477 | 57.52380952380953 | 68.9047619047619 | 67.3 |
| 100 | None | None | None | None |
| 105 | None | None | None | None |
| 110 | None | None | None | None |
| 115 | None | None | None | None |
| 120 | None | None | None | None |
| 125 | None | None | None | None |CURINGA
TE-2014-15
Irrigation termination
Direct seeding (Sowing)
Days to 50% Flowering
Harvesting
Re-Watering
Vegetative Stage
Normal Irrigation
Data collection

## Slide 4
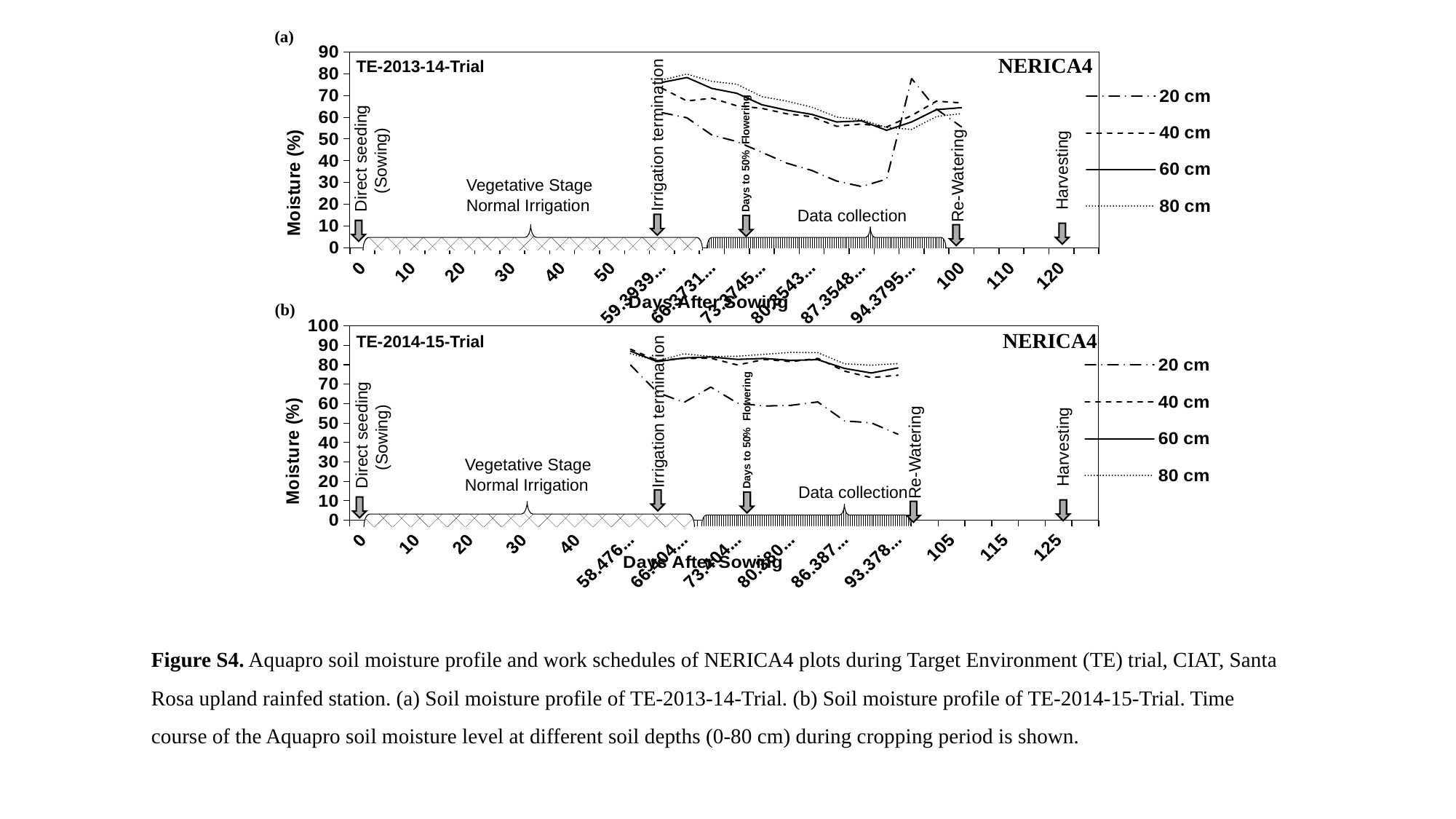

(a)
### Chart
| Category | 20 cm | 40 cm | 60 cm | 80 cm |
|---|---|---|---|---|
| 0 | None | None | None | None |
| 5 | None | None | None | None |
| 10 | None | None | None | None |
| 15 | None | None | None | None |
| 20 | None | None | None | None |
| 25 | None | None | None | None |
| 30 | None | None | None | None |
| 35 | None | None | None | None |
| 40 | None | None | None | None |
| 45 | None | None | None | None |
| 50 | None | None | None | None |
| 55 | None | None | None | None |
| 59.393958333334012 | 62.08333333333334 | 73.3333333333333 | 76.0 | 77.0833333333333 |
| 62.414467592592672 | 59.75 | 67.5 | 78.25 | 79.8333333333333 |
| 66.373101851851914 | 51.83333333333334 | 68.75 | 73.25 | 76.5 |
| 70.36629629629897 | 48.83333333333334 | 65.25 | 71.0 | 75.16666666666667 |
| 73.374560185184208 | 43.91666666666662 | 64.0833333333333 | 65.75 | 69.5 |
| 77.380335648151203 | 38.91666666666662 | 61.58333333333334 | 63.25 | 67.4166666666667 |
| 80.354305555556437 | 35.58333333333334 | 60.25 | 61.33333333333334 | 64.66666666666667 |
| 84.367361111108039 | 30.66666666666667 | 55.83333333333334 | 57.83333333333334 | 60.08333333333334 |
| 87.354803240741603 | 28.16666666666667 | 56.91666666666662 | 58.33333333333334 | 58.83333333333334 |
| 91.364594907405262 | 31.58333333333331 | 55.5 | 54.0 | 55.33333333333334 |
| 94.379513888889051 | 77.75 | 60.75 | 57.83333333333334 | 54.41666666666662 |
| 97.353472222224752 | 63.75 | 67.4166666666667 | 63.5 | 60.33333333333334 |
| 100 | 55.58333333333334 | 66.5833333333333 | 64.4166666666667 | 61.66666666666664 |
| 105 | None | None | None | None |
| 110 | None | None | None | None |
| 115 | None | None | None | None |
| 120 | None | None | None | None |
| 125 | None | None | None | None |NERICA4
TE-2013-14-Trial
Irrigation termination
Direct seeding (Sowing)
Days to 50% Flowering
Harvesting
Re-Watering
Vegetative Stage
Normal Irrigation
Data collection
(b)
### Chart
| Category | 20 cm | 40 cm | 60 cm | 80 cm |
|---|---|---|---|---|
| 0 | None | None | None | None |
| 5 | None | None | None | None |
| 10 | None | None | None | None |
| 15 | None | None | None | None |
| 20 | None | None | None | None |
| 25 | None | None | None | None |
| 30 | None | None | None | None |
| 35 | None | None | None | None |
| 40 | None | None | None | None |
| 45 | None | None | None | None |
| 58.476249999999709 | 79.87499999999999 | 88.0 | 87.0 | 85.75 |
| 63.410150462965248 | 65.75 | 82.25 | 81.62499999999999 | 82.0 |
| 66.404374999998254 | 60.625 | 83.25 | 83.5 | 85.62499999999999 |
| 70.421261574076198 | 68.5 | 83.37499999999999 | 84.0 | 84.25 |
| 73.404398148144509 | 60.25 | 79.87499999999999 | 82.75 | 84.37499999999999 |
| 77.376099537039366 | 58.75 | 82.75 | 83.25 | 85.37499999999999 |
| 80.380416666666846 | 59.125 | 81.62499999999999 | 82.25 | 86.37499999999999 |
| 83.368113425924093 | 60.875 | 83.25 | 82.62499999999999 | 86.25 |
| 86.387847222220415 | 51.125 | 76.75 | 78.12499999999999 | 80.5 |
| 90.387789351851055 | 50.125 | 73.37499999999999 | 75.75 | 79.75 |
| 93.378067129626245 | 44.25 | 74.62499999999999 | 78.37499999999999 | 80.62499999999999 |
| 100 | None | None | None | None |
| 105 | None | None | None | None |
| 110 | None | None | None | None |
| 115 | None | None | None | None |
| 120 | None | None | None | None |
| 125 | None | None | None | None |NERICA4
TE-2014-15-Trial
Irrigation termination
Direct seeding (Sowing)
Days to 50% Flowering
Harvesting
Re-Watering
Vegetative Stage
Normal Irrigation
Data collection
Figure S4. Aquapro soil moisture profile and work schedules of NERICA4 plots during Target Environment (TE) trial, CIAT, Santa Rosa upland rainfed station. (a) Soil moisture profile of TE-2013-14-Trial. (b) Soil moisture profile of TE-2014-15-Trial. Time course of the Aquapro soil moisture level at different soil depths (0-80 cm) during cropping period is shown.

## Slide 5
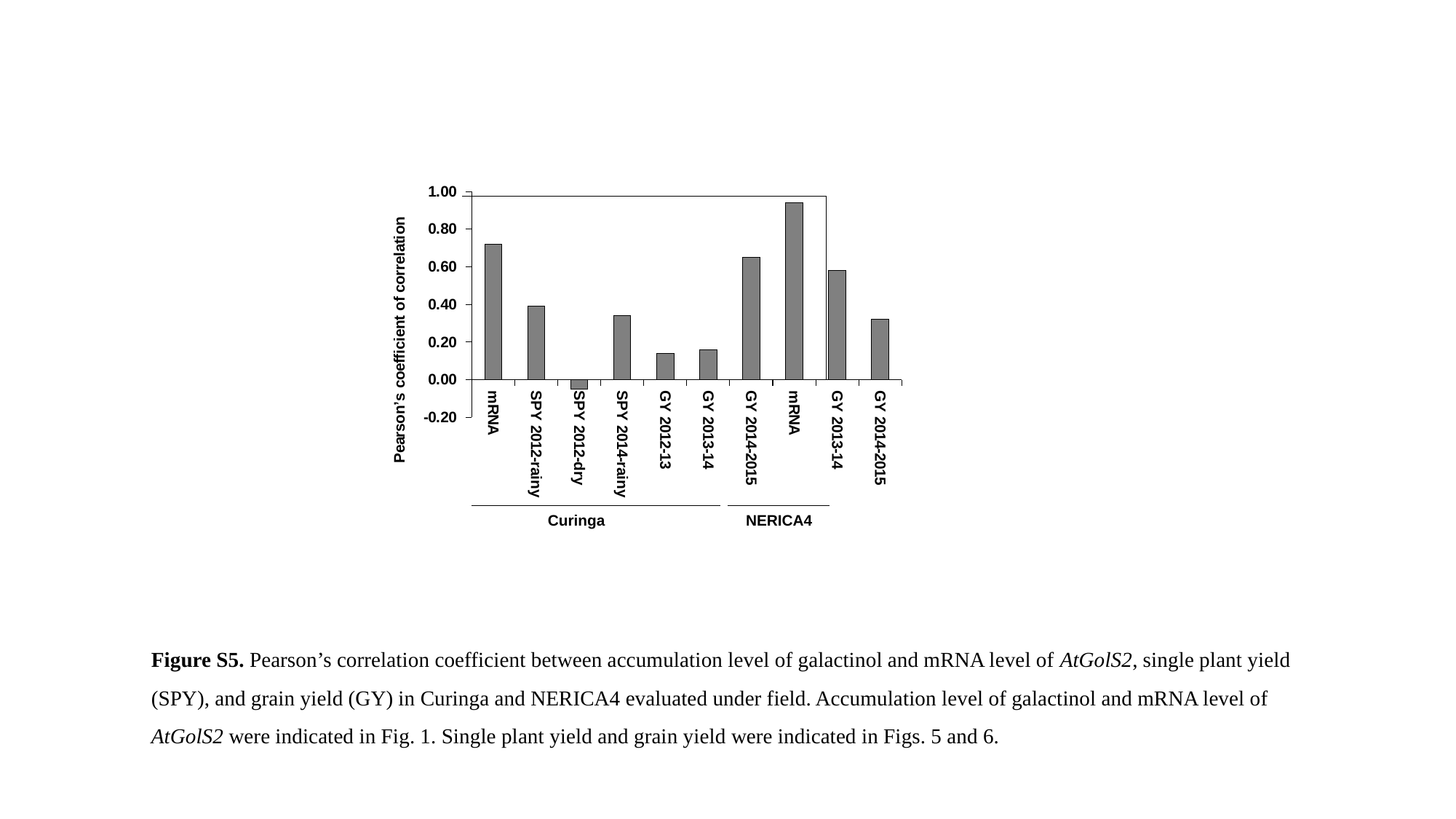

### Chart
| Category | |
|---|---|
| mRNA | 0.72 |
| SPY 2012-rainy | 0.39 |
| SPY 2012-dry | -0.05 |
| SPY 2014-rainy | 0.34 |
| GY 2012-13 | 0.14 |
| GY 2013-14 | 0.16 |
| GY 2014-2015 | 0.65 |
| mRNA | 0.94 |
| GY 2013-14 | 0.58 |
| GY 2014-2015 | 0.32 |Curinga
NERICA4
Figure S5. Pearson’s correlation coefficient between accumulation level of galactinol and mRNA level of AtGolS2, single plant yield (SPY), and grain yield (GY) in Curinga and NERICA4 evaluated under field. Accumulation level of galactinol and mRNA level of AtGolS2 were indicated in Fig. 1. Single plant yield and grain yield were indicated in Figs. 5 and 6.
