# Supplementary material for: Overexpression of an Arabidopsis thaliana galactinol synthase gene improves drought tolerance in transgenic rice and increased grain yield in the field
Source: Plant Biotechnol J. 2017 May 3;15(11):1465–77. doi: 10.1111/pbi.12731 (PMC5633756; doi:10.1111/pbi.12731)
Supplement: Supplementary file 2 — Table S1 Agronomic data capture from 2012 well‐watered paddy field trial at CIAT, Palmira, using Ubi:AtGolS2 Curinga lines. Table S2 Agronomic data capture from rainout‐shelter trial (2012‐rainy‐MDSE‐Trial‐1, 2012‐dry‐MDSE‐Trial‐2, and 2014‐rainy‐Trial) at CIAT, Palmira using Ubi:AtGolS2 Curinga transgenic lines. Table S3 Agronomic data capture from Santa Rosa target Environment (TE) upland trial using Ubi:AtGolS2 Curinga lines. Table S4 The survival rates of NT NERICA4, Ubi:AtGolS2 NERICA4 lines under drought stress. Table S5 Agronomic data capture from Santa Rosa target environment (TE) trial using Ubi:AtGolS2 NERICA4 lines. Table S6 Primer sequences used in this study. [file PBI-15-1465-s001.docx]

**Table S1**. Agronomic Data capture from 2012 well water paddy field trial at CIAT, Palmira, using *Ubi:AtGolS2* Curinga events.

| **Event** | **DFL(100%)** | **PH** | **TN** | **PN** | **SPY(g)** | **GYB (Kg/Ha)** |
| --- | --- | --- | --- | --- | --- | --- |
| **2580** | 84.7 ± 1.3 | 104.3 ± 0.5 | 17.0 ± 1.7 | 15.8 ± 1.3 | 31.5 ± 0.5 | 11750.1 ± 814.0 |
| **2590** | 82.3 ± 0.9 | 112.7 ± 1.9 | 16.8 ± 1.0 | 15.7 ± 0.7 | 30.9 ± 1.7 | 10801.2 ± 793.4 |
| **2783** | 82.7 ± 0.7 | 104.1 ± 1.2 **^-^** | 18.7 ± 0.3 | 16.2 ± 0.5 | 31.9 ± 0.6 | 12015.7 ± 596.8 |
| **2822** | 84.7 ± 0.7 | 109.7 ± 4.8 | 17.0 ± 0.8 | 15.6 ± 0.4 | 32.2 ± 1.5 | 11420.0 ± 919.0 |
| **3020** | 82.3 ± 0.3 | 105.4 ± 0.9 **^-^** | 16.8 ± 0.9 | 15.9 ± 0.3 | 32.2 ± 4.0 | 11969.8 ± 928.2 |
| **3025** | 84.0 ± 1.2 | 116.2 ± 2.6 | 16.6 ± 0.4 | 15.7 ± 0.8 | 30.4 ± 1.8 | 12303.0 ± 381.4 |
| **3214** | 83.7 ± 1.5 | 108.2 ± 3.4 | 16.8 ± 0.7 | 16.2 ± 0.4 | 30.6 ± 2.6 | 12824.0 ± 609.5 |
| **Curinga NT** | 84.4 ± 0.2 | 108.9 ± 0.9 | 18.1 ± 0.4 | 15.4 ± 0.4 | 30.6 ± 0.3 | 12984.1 ± 1204.3 |
| ***P*-value** | 0.37 | 0.04 | 0.68 | 0.98 | 0.99 | 0.62 |

DFL, Days to 100% Flowering; PH, plant height (cm); TN, tiller number; PN, panicle number (Fully emerged); SPY, single plant grain yield (g); GYB, grain yield from the bulk (Kg/Ha). Each grain yield and yield related parameter value represents the mean ± SE (n=9-12); Flowering data based on whole plot performance and represents the mean ± SE (n = 3) from three replications. *P*-values were calculated by one-way ANOVA.

**Table S2**. Agronomic data capture from rainout-shelter trial (2012-rainy-MDSE-Trial-1, 2012-dry-MDSE-Trial-2, and 2014-rainy-Trial) at CIAT, Palmira using *Ubi:AtGolS2* Curinga transgenic events.

| **MDSE Trial** | **Event** | **PH** | **PN** | **BM** | **DFL/DFF** | **LR** | **LD** | **SPAD PS** | **DRS** |
| --- | --- | --- | --- | --- | --- | --- | --- | --- | --- |
| 2012-I | 2580 | 89.0 ± 2.1 ab | 10.3 ± 0.9 | 17.1 ± 0.5 b | 77.7 ± 0.7 ab | 1.7 ± 0.7 a | 1.0 ± 0.0 a |  | 2.0 ± 0.6 a |
|  | 2590 | 86.3 ± 3.5 ab | 10.3 ± 0.3 | 16.1 ± 0.6 ab | 78.3 ± 0.7 ab | 1.7 ± 0.7 a | 1.7 ± 0.7 ab |  | 2.0 ± 0.6 a |
|  | 2783 | 80.7 ± 2.3 a | 10.2 ± 1.1 | 16.1 ± 0.9 ab | 81.0 ± 0.6 c | 2.3 ± 0.7 a | 2.3 ± 0.7 ab |  | 2.0 ± 0.0 a |
|  | 2822 | 95.7 ± 3.5 ab | 8.6 ± 0.6 | 14.6 ± 1.1 ab | 78.3 ± 0.7 ab | 5.0 ± 0.0 bc | 5.0 ± 0 cd |  | 2.0 ± 0.6 a |
|  | 3020 | 95.0 ± 6.4 ab | 12.7 ± 1.7 | 17.2 ± 0.8 b | 79.0 ± 0.0 abc | 3.0 ± 0.0 ab | 3.0 ± 0.0 abc |  | 2.0 ± 0.6 a |
|  | 3025 | 90.3 ± 0.7 ab | 8.2 ± 1.5 | 12.9 ± 1.4 ab | 77 ± 0.0 a | 3.7 ± 0.7 abc | 3.7 ± 0.7 bc |  | 2.0 ± 0.6 a |
|  | 3214 | 90.3 ± 0.7 ab | 8.2 ± 1.5 | 15.3 ± 0.3 ab | 79.3 ± 0.3 bc | 3.7 ± 0.7 abc | 3.7 ± 0.7 ab |  | 2.0 ± 0.6 a |
|  | Curinga NT | 96.6 ± 2.3 b | 7.9 ± 0.3 | 12.6 ± 0.8 a | 81.1 ± 0.2 c | 6.1 ± 0.2 c | 6.1 ± 0.2 d |  | 5.0 ± 0.0 b |
|  | *P*-value | 0.04 | 0.11 | 0.01 | <0.01 | <0.01 | <0.01 |  | <0.01 |
| 2012-II | 2580 | 70.5 ± 0.5 | 11.2 ± 0.3 b | 20. 6 ± 0.7 b | 79.0 ± 0.0 a | 3.0 ± 0.0 a | 3.0 ± 0.0 ab | 28.8 ± 0.4 d | 2.0 ± 0.6 a |
|  | 2590 | 73.3 ± 1.4 | 11.8 ± 0.3 b | 21.3 ± 0.9 b | 79.3 ± 0.3 a | 3.0 ± 0.0 a | 2.3 ± 0.7 a | 23.4 ± 0.8 c | 2.0 ± 0.6 a |
|  | 2783 | 73.9 ± 0.8 | 10.5 ± 0.4 b | 17.3 ± 0.4 ab | 80.3 ± 0.3 ab | 4.3 ± 0.7 ab | 3.0 ± 0.0 ab | 13.9 ± 0.2 a | 2.0 ± 0.0 a |
|  | 3020 | 72.7 ± 2.3 | 11.4 ± 0.3 b | 15.9 ± 2.5 ab | 80.0 ± 1.0 ab | 3.7 ± 0.7 a | 3.7 ± 0.7 ab | 16.2 ± 0.9 ab | 2.0 ± 0.6 a |
|  | 3025 | 72.9 ± 0.8 | 10.3 ± 0.1 b | 14.8 ± 0.9 a | 80.0 ± 1.0 ab | 3.7 ± 0.7 a | 5.7 ± 0.7 b | 16.3 ± 1.1 ab | 2.0 ± 0.6 a |
|  | 3214 | 73.3 ± 0.1 | 10.3 ± 0.3 b | 14.0 ± 0.2 a | 80.3 ± 0.9 ab | 3.0 ± 0.0 a | 5.0 ± 1.2 ab | 18.8 ± 1.2 b | 2.0 ± 0.6 a |
|  | Curinga NT | 73.5 ± 0.9 | 7.1 ± 0.5 a | 18.1 ± 0.5 ab | 82.6 ± 0.3 b | 6.6 ± 0.7 b | 5.3 ± 0.2 ab | 23.4 ± 1.2 c | 5.0 ± 0.0 b |
|  | *P*-value | 0.52 | <0.01 | <0.01 | 0.04 | <0.01 | <0.01 | <0.01 | <0.01 |
| 2014 | 2580 | 73.8 ± 0.7 | 7.6 ± 0.2 | 9.2 ± 1.7 | 78.7 ± 0.3 a | 2.3 ± 0.7 a | 2.3 ± 0.7 a | 36.3 ± 1.8 ab | 1.7 ± 0.7 |
|  | 2590 | 72.2 ± 3.1 | 7.5 ± 0.5 | 7.4 ± 1.8 | 77.7 ± 1.3 a | 2.3 ± 0.7 a | 1.7 ± 0.7 a | 39.0 ± 1.0 b | 1.7 ± 0.7 |
|  | 2783 | 72.8 ± 9.0 | 5.4 ± 1.2 | 8.3 ± 1.00 | 81.3 ± 0.7 ab | 3.7 ± 0.7 a | 3.0 ± 0.0 a | 37.0 ± 2.3 ab | 2.3 ± 1.3 |
|  | 3020 | 69.9 ± 5.4 | 5.4 ± 1.0 | 6.5 ± 1.8 | 78.0 ± 1.5 a | 3.7 ± 0.7 a | 2.3 ± 0.7 a | 35.7 ± 0.6 ab | 1.7 ± 0.7 |
|  | 3214 | 71.1 ± 9.2 | 6.3 ± 1.5 | 7.6 ± 1.8 | 77.0 ± 1.0 a | 3.0 ± 0.0 a | 2.3 ± 0.7 a | 34.8 ± 1.1 ab | 1.7 ± 0.7 |
|  | Curinga NT | 72.8 ± 0.5 | 6.6 ± 0.5 | 7.3 ± 0.4 | 83.7 ± 0.3 b | 6.7 ± 0.3 b | 5.7 ± 0.7 b | 31.4 ± 1.2 a | 4.3 ± 0.7 |
|  | *P*-value | 1.00 | 0.42 | 0.87 | <0.01 | <0.01 | <0.01 | 0.05 | 0.21 |

PH, plant height (cm); PN, Panicle number (fully emerged); BM, Biomass; DFL, Days to 50% Flowering; DFF, Days to First Flowering (MDSE-2014 trial alone) ; LR, Leaf Rolling; LD, Leaf Drying; SPAD PS, SPAD value at peak stress; DRS, Drought Recovery Score. Each grain related parameter value represents the mean ± SE (n = 9-24); Flowering and leaf rolling, leaf drying and recovery score value based on whole plot performance and represents the mean ± SE (n = 3). SPAD value represents the mean ± SE (n = 9), 3 individual plants per replication. *P*-values were calculated by one-way ANOVA. Different letters in each column denote significant differences at *P*<0.05 by Tukey-Kramer method.

**Table S3**. Agronomic data capture from Santa Rosa target Environment (TE) upland trial using *Ubi:AtGolS2* Curinga events.

| **Season/Year** | **Event** | **DFL (50%)** | **PH** | **TN** | **PN** | **LR** | **PL** |
| --- | --- | --- | --- | --- | --- | --- | --- |
| TE_2012-2013 | 2580 | 70.3 ± 0.3 ab | 100.5 ± 0.7 | 10.2 ± 0.1 bc | 8.9 ± 0.1 ab |  |  |
|  | 2590 | 70.0 ± 0.0 ab | 104.1 ± 0.5 | 11.2 ± 0.2 c | 10.7 ± 0.4 b |  |  |
|  | 2783 | 68.7 ± 0.7 a | 99.2 ± 1.2 | 10.0 ± 0.6 bc | 9.7 ± 1.4 ab |  |  |
|  | 3020 | 72.3 ± 1.5 bc | 104.9 ± 1.0 | 7.6 ± 0.7 a | 6.5 ± 0.4 a |  |  |
|  | 3214 | 69.0 ± 0.6 a | 103.3 ± 2.5 | 8.9 ± 0.8 abc | 8.1 ± 0.6 ab |  |  |
|  | Curinga NT | 74.5 ± 0.3 c | 103.9 ± 0.6 | 8.2. ± 0.4 ab | 7.2 ± 0.6 a |  |  |
|  | *P*-value | <0.01 | 0.05 | <0.01 | <0.01 |  |  |
| TE_2013-2014 | 2580 | 79.0 ± 1.2 | 86.1 ± 1.1 |  | 10.1 ± 1.4 | 1.0 ± 0.0 | 22.2 ± 0.6 b |
|  | 2590 | 77.0 ± 1.0 | 87.7 ± 1.1 |  | 10.2 ± 1.6 | 1.0 ± 0.0 | 22.6 ± 1.2 b |
|  | 2783 | 80.0 ± 0.9 | 89.4 ± 1.2 |  | 9.3 ± 0.2 | 2.3 ± 0.7 | 23.9 ± 0.2 b |
|  | 3020 | 78.7 ± 1.7 | 86.8 ± 1.1 |  | 7.7 ± 1.0 | 2.3 ± 0.7 | 21.8 ± 0.4 b |
|  | 3214 | 77.7 ± 1.5 | 88.3 ± 0.6 |  | 6.8 ± 0.9 | 3.7 ± 0.7 | 16.9 ± 1.8 a |
|  | Curinga NT | 79.6 ± 0.8 | 90.2 ± 1.9 |  | 6.2 ± 0.2 | 2.7 ± 0.7 | 21.6 ± 0.1 b |
|  | *P*-value | 0.59 | 0.23 |  | 0.06 | 0.04 | <0.01 |
| TE_2014-2015 | 2580 | 80.0 ± 1.5 | 103.7 ± 0.4 | 10.6 ± 0.7 | 8.3 ± 0.7 | 3.0 ± 0.0 |  |
|  | 2590 | 76.3 ± 1.5 | 99.7 ± 1.2 | 10.1 ± 0.6 | 8.1 ± 0.7 | 3.0 ± 0.0 |  |
|  | 3020 | 75.0 ± 1.2 | 100.1 ± 1.5 | 9.1 ± 0.6 | 7.0 ± 0.3 | 3.0 ± 0.0 |  |
|  | 3214 | 77.3 ± 0.9 | 100.7 ± 1.8 | 9.8 ± 1.0 | 7.4 ± 0.6 | 1.7 ± 0.7 |  |
|  | Curinga NT | 80.2 ± 0.3 | 105.6 ± 2.0 | 7.9 ± 0.1 | 6.2 ± 0.2 | 2.1 ± 0.2 |  |
|  | *P*-value | 0.04 | 0.07 | 0.12 | 0.11 | 0.03 |  |

DFL, Days to 50% Flowering; PH, plant height (cm); TN, tiller number; PN, panicle number (Fully emerged); LR, Leaf Rolling; PL, Panicle Length (cm). Each grain yield related parameter value represents the mean ± SE (n = 15-24); Flowering and leaf rolling value based on whole plot performance and represents the mean ± SE (n = 3), 3 individual plants per replication. *P*-values were calculated by one-way ANOVA. Different letters in each column denote significant differences at *P*<0.05 by Tukey-Kramer method.

**Table S4.** The survival rates of NT NERICA, *Ubi:AtGolS2* NERICA4 events under drought stress.

| **Event** | **No. plants tested** | **No. plants survived** | **% survival** |
| --- | --- | --- | --- |
| 1575 | 140 | 43 | 30.7^c^ |
| 1577 | 142 | 39 | 27.5^bc^ |
| 2054 | 145 | 38 | 26.2^bc^ |
| 2344 | 148 | 51 | 34.5^c^ |
| 2361 | 139 | 23 | 16.5^ab^ |
| 2362 | 140 | 23 | 16.4^ab^ |
| NERICA4 NT | 148 | 17 | 11.5^a^ |

Different letters denote significant differences at *P*<0.05 by Tukey’s test

**Table S5**. Agronomic data capture from Santa Rosa target environment (TE) trial using *Ubi:AtGolS2* NERICA4 events.

| **TE Trial** | **Event** | **PH** | **PN** | **PL** | **DFL (50%)** | **LR** | **LD** |
| --- | --- | --- | --- | --- | --- | --- | --- |
| TE 2013-14 | 1577 | 89.3 ± 2.4 a | 5.9 ± 0.5 ab | 23.3 ± 0.6 | 67.5 ± 0.9 | 2.0 ± 0.6 ab | 2.0 ± 0.6 ab |
|  | 1577 | 91.9 ± 2.6 b | 7.3 ± 0.1 b | 22.8 ± 1.1 | 68.7 ± 0.3 | 1.1 ± 0.1 ab | 1.1 ± 0.1 a |
|  | 2054 | 84.3 ± 2.9 ab | 4.7 ± 0.3 a | 21.7 ± 0.7 | 68.3 ± 1.2 | 1.7 ± 0.7 ab | 1.7 ± 0.7 ab |
|  | 2344 | 87.8 ± 3.1 ab | 5.7 ± 0.6 ab | 23.0 ± 0.7 | 65.3 ± 0.9 | 2.1 ± 0.6 ab | 2.1 ± 0.6 ab |
|  | 2361 | 79.1 ± 0.5 a | 7.0 ± 0.4 b | 20.1 ± 0.2 | 65.7 ± 2.2 | 1.0 ± 0.0 a | 0.9 ± 0.1 a |
|  | 2362 | 88.8 ± 2.2 ab | 5.7 ± 0.7 ab | 23.3 ± 0.6 | 68.0 ± 0.0 | 1.2 ± 0.2 ab | 1.3 ± 0.2 a |
|  | NERICA4 NT | 87.6 ± 0.5 ab | 4.7 ± 0.1 a | 22.9 ± 0.4 | 67.5 ± 0.4 | 3.4 ± 0.4 b | 3.4 ± 0.4 b |
|  | *P*-value | 0.03 | <0.01 | 0.10 | 0.26 | 0.02 | 0.02 |
| TE 2014-15 | 1575 | 109.8 ± 2.3 | 6.6 ± 1.1 b |  | 68.0 ± 0.0 b | 1.0 ± 0.0 a |  |
|  | 1577 | 108.8 ± 2.5 | 6.7 ± 0.1 b |  | 64.7 ± 0.9 a | 1.0 ± 0.0 a |  |
|  | 2344 | 110.6 ± 1.4 | 5.9 ± 0.3 ab |  | 67.0 ± 0.0 ab | 1.0 ± 0.0 a |  |
|  | 2361 | 108.9 ± 2.6 | 5.5 ± 0.2 a |  | 66.3 ± 0.7 ab | 3.0 ± 0.0 b |  |
|  | 2362 | 111.0 ± 0.9 | 6.8 ± 0.3 b |  | 67.7 ± 0.3 b | 2.3 ± 0.7 ab |  |
|  | NERICA4 NT | 115.8 ± 0.1 | 5.9 ± 0.2 ab |  | 66.6 ± 0.4 ab | 3.2 ± 0.2 b |  |
|  | *P*-value | 0.17 | 0.01 |  | <0.01 | <0.01 |  |

PH, plant height (cm); PN, panicle number (Fully emerged); PL, Panicle Length; FL, Days to 50% Flowering ; LR, Leaf Rolling; LD, Leaf Drying. Each grain yield related parameter value represents the mean ± SE (n = 15-18); Flowering, leaf rolling and leaf drying value based on whole plot performance and represents the mean ± SE (n = 3) from three replications, 3 individual plants per replication. *P*-values were calculated by one-way ANOVA. Different letters in each column denote significant differences at *P*<0.05 by Tukey-Kramer method.

**Table S6.** Primer sequences used in this study

|  | | |
| --- | --- | --- |
| Experiment | Primer name | Primer sequence (5' - 3') |
| Amplificaiton of fragments of *AtGolS2* gene | AtGolS2 F1 | AACCTCTTGGAGACGGTCAA |
|  | AtGolS2 R1 | AGATGGAGCTTTGACATACTG |
| Amplification of fragment of HPT gene | HPT F1 | TCGTGCTTTCAGCTTCGATG |
|  | HPT R1 | TCCATCACAGTTTGCCAGTG |
| qRT-PCR | AtGolS2-F01 | ACTTCAACGCCGGTATGTTC |
|  | AtGolS2-R01 | AAAGCATTGCCAAGACCAAG |
|  | OsUbi-qF01 | GGACACAATGATTAGGGATCA |
|  | OsUbi-qR01  OsLEA3-qF  OsLEA3-qR  ICL-qF  ICL-qR  OsNac6-qF  OsNac6-qR | GTGGTGGCCAGTAAGTCCTC  GAGTGAGCAGGTGAAGAGCA  GTGGCAGAGGTGTCCTTGTT  CAGGGTGGCATCTCCTCTAC  TGAGCCCTTGAACTGCTCTT  AGAAGACCAACTGGATCATGCA  CGGCACAGCACCCAATC |
